# Visual acuity in controls and patients measured with Maxwellian view and a 3 mm pupil: Examining potential effects of inherent and induced aberrations

**DOI:** 10.1371/journal.pone.0352879

**Published:** 2026-06-29

**Authors:** Vamsi Parimi, Ann E. Elsner, Christopher A. Clark, Brett J. King

**Affiliations:** 1 School of Optometry, Indiana University, Bloomington, Indiana, United States of America; 2 Aeon Imaging, LLC, Bloomington, Indiana, United States of America; Aravind Eye Care System, INDIA

## Abstract

We sought to improve the measurement of visual acuity (VA) by reducing the effect of unwanted optical artifacts in patients with retinal disease. We evaluated the effects of age and of inherent and induced aberrations on VA for controls and patients with changes to the posterior segment, anterior segment, or both. We tested VA in 30 controls and 17 patients, using Maxwellian view and a 3 mm pupil. This method reduces wavefront aberrations and allows high spatial frequency information to be transmitted at a constant retinal illuminance. We reduced glare, crowding, and fixation errors by projecting a single letter E in a small field size. We corrected only defocus, then determined the extent to which VA and standard deviation (SD) were influenced by age and inherent wavefront aberrations (astigmatism, spherical aberration, and root mean square error higher order aberrations (RMSHO)), as well as by induced aberrations (spherical aberrations and coma). Optical coherence tomography (OCT) provided central macular thickness (CMT), which was compared to wavefront and VA results to determine the effect of retinal status. The use of Maxwellian view and a 3 mm pupil reduced aberrations, but the association between VA and inherent astigmatism (*p* = 0.011) was significant for controls, although not for patients. For controls, RMSHO significantly increased with age (*p* = 0.014), despite no impact on VA. Induced aberrations, which were as large as with 5 mm pupils, significantly worsened mean VA in controls (*p* < 0.001) and patients (*p* < 0.001), more for positive spherical aberrations than negative. Using Maxwellian view and a 3 mm pupil reduced optical aberrations and their effects on VA, important in managing patients with retinal disease. Only residual astigmatism was related to poorer VA.

## Introduction

High contrast, high light level VA is the key primary measure of visual function that serves as a critical metric in routine clinical practice as well as clinical trials [1–6]. One goal of VA testing is to assess the health of the retina, which differs considerably from the goal of assessing visual impairment as a predictor for difficulties with activities of daily living and of mortality [7]. For determining the effectiveness of treatments for retinal disease, anterior segment changes should be minimized, as they lead to unwanted noise in the measurements. In contrast, when assessing functional vision, the changes in both anterior segment and posterior segment should be included. Despite these different goals, the methodology used differs little, with a chief difference being that monocular testing is used in the retinal disease treatment trials or patient management, but binocular testing for the assessment of visual impairment [3,5,7,8].

The standard clinical VA tests involve direct viewing of printed charts or digital displays. This method, also known as Newtonian viewing, does not control the fixation location. Whether using a chart or digital display, even when incorporated into goggles or a headset, these common tests suffer from inherent limitations due to variability in pupil size [9,10], which affects both retinal illuminance and the magnitudes of ocular aberrations. VA is poorer when the pupil is sufficiently small to significantly reduce retinal illuminance [11,12]. VA can also decrease when the pupil size is sufficiently large in a patient with a significant increase in the magnitude of ocular aberrations with pupil size [13–17]. Aberrations generally increase with increasing age, mainly for larger pupil sizes, with the amount varying with type of aberration [13–17]. Other optical artifacts impacting VA that increase with increasing age include tear film instability [18,19], lenticular opacities [20–22], and intraocular scatter [23]. The conventional use of pinhole for clinical VA measurements greatly reduces the effects of aberrations and unwanted scatter. However, for a small entrance pupil there are significant diffraction effects that lead to decreased transmission of higher spatial frequencies, which along with a reduction in retinal illuminance or misplacement of the pinhole [24] can degrade VA [11,25].

Additional limitations of conventional VA testing include the effects of light scatter and halos, letter crowding, and the inability to quantify within-subject variability [23,26,27]. These factors, along with the above mentioned optical factors, contribute to the significant decline with age of other visual functions such as contrast sensitivity, low-contrast VA, and glare sensitivity [28,29]. High-contrast VA has a relatively minimal decline with age [29,30], and therefore is commonly selected to assess differences in retinal health in patients. Nevertheless, the variability of VA measurements for individual patients remains a source of concern for the consensus on visual evaluation in retinal disease management [22].

To minimize the optical effects on VA measurements, we previously developed the potential vision tester (PVT), which projects a visual stimulus through a fixed 3 mm pupil using a Maxwellian view configuration, described in more detail previously [27,31]. The PVT prototypes are optimized for a 3 mm pupil, guided by previous findings. Both the magnitude and the phase of the transfer through the pupil must be considered. The decrease in the magnitude of the target contrast increases with spatial frequency, which impacts the image of the stimulus on the retina. Although the human eye is considered essentially diffraction-limited for 2–3 mm diameter pupils [32], the wider pupil diameters in this range allow high spatial frequency information to pass. The contrast ratio measured with psychophysics at 40–50 cpd is better for a 2.8 mm pupil than a 2 or 3.8 mm pupil [11]. Similarly, a 3.0 mm pupil provides the narrowest line spread functions with a reflectometry method [25]. The shifts in phase also lead to decreased contrast, and even ghost images, with information at different spatial frequencies in the same letter having more negative or positive contrast. Phase shift has long been known to depend on pupil size. Phase transfer as a function of spatial frequency significantly reverses direction for pupil sizes larger than 3 mm, reaching large shifts in phase above 30 cpd [32]. Both phase reversals and the large phase shifts at high spatial frequency likely impact VA. For these reasons, when aberrations beyond sphere and cylinder remain uncorrected, a pupil size of 3 mm has been selected [33].

A common method to control pupil size while also achieving precise control of the retinal image focus and illuminance is achieved by using Maxwellian view. Imaging a light source in the eye’s pupil, instead of looking at it directly as in Newtonian view, has since been applied widely and is known as Maxwellian view [34,35]. The strength of a Maxwellian view system is that properties of the target, such as focus, size, and shape, can be controlled independently from retinal illuminance [34] while retaining the key design elements. In the current system [27] there is careful separation of pupil planes and retinal planes, with the final exit pupil plane of the instrument imaged onto the pupil of the eye, while the retinal plane is focused by the refractive elements of the eye and ancillary optics that are placed in pupil conjugate planes.

The use of a 3 mm pupil in the Maxwellian view system further reduces ocular aberrations while limiting diffraction effects found with smaller pupil sizes [25]. In the present study, the main characterization of optical factors is in terms of wavefront aberrations, which are defined as the deviations of light from the ideal path [36] and are typically characterized as Zernike polynomials [37]. Simulations of the distortions to letters from specific wavefront aberrations have been developed and verified by measurements [33]. Commercial aberrometers and wavefront-based autorefractors display distorted letters simulated from wavefront measurements, often as a function of pupil size [13].

By reducing the measurement area to include only a 3 mm pupil, we found that the only wavefront aberrations that increased with age included vertical astigmatism, horizontal coma, spherical aberration, and vertical quadrafoil [13]. The magnitude of these aberrations was significantly smaller for the 3 mm pupil compared to a 5 mm pupil, as expected.

While our previous research on wavefront aberrations characterized control subjects for a wide range of ages, our VA studies to date have included smaller numbers of subjects and relatively small inherent wavefront errors [27]. A wider range of ages and larger wavefront errors are needed to determine their effects on VA and whether using Maxwellian view and a 3 mm pupil can reduce unwanted optical errors so that age norms are not needed and variability of VA is more likely due to the health of the retina. In the current study, we evaluated the VA measurements using the PVT in a diverse cohort of subjects, including controls over a wide range of age and patients with posterior segment and/or anterior segment issues representative of patients with retinal disease in a clinical trial population and in real world clinical care. We tested the effects of inherent aberrations on VA when only the defocus was corrected. The goal of our study was to determine whether an instrument to measure VA that reports on retinal disease must have the added components and procedural elements required to correct for the inherent astigmatism. Specifically, we tested whether using Maxwellian view and a pupil of only 3 mm, which we previously showed significantly reduced aberrations [13], is sufficient to provide VA measurements that do not vary with inherent astigmatism. In our initial study on VA, the subjects did not have sufficiently high astigmatism to evaluate the effect [27]. This also has direct clinical relevance to patient populations who are not corrected for astigmatism, whether due to changes with aging [13], the result of IOL surgery [38] including not opting for the added expense of toric IOLs, or the substantial number of contact lens wearers who do not wear toric lenses [39]. Further, understanding the effect on VA of various wavefront aberrations and using a method that provides both mean and SD can provide a clearer interpretation of mean and variability of VA in clinical studies. This is urgently needed given the societal and environmental impact of providing intravitreal injections estimated to exceed 15,000,000 per year in the US alone [40]. It is especially important for trials with durations in years and patients managed for exudation, particularly for patients who have changes over time in lens and capsule status or corneal changes that are unrelated to retinal health.

Further, we evaluated the impact on VA measurements when using experimentally induced aberrations of the magnitude that is found with large pupil sizes in this population. We evaluated the impact of age for both inherent and induced aberrations. We studied potential changes in relation to CMT as seen on OCT, which can indicate disruption of retinal layers that could impact either wavefront measurements or VA. Previous studies have indicated that wavefront errors are affected for patient groups likely to have early cataract, i.e., patients with diabetes or retinitis pigmentosa [41,42] as well as trends in other groups [43]. In addition to defining instrumentation designed to improve the measurement of VA, the wavefront aberrations approach has implications for understanding the VA found with intentionally induced changes in pupil size, e.g., reduced pupil size for presbyopia treatment [44] and increased pupil size for myopia treatment [45].

The aim of this study was to investigate whether the effects of inherent and induced wavefront aberrations on VA were still important when reducing wavefront aberrations by using a Maxwellian view configuration with a 3 mm pupil and correction limited to defocus alone. The study indicated for both the controls and patients tested, that higher order wavefront aberrations have a negligible impact on VA under these conditions. The primary optical factor affecting VA was residual uncorrected astigmatism of the magnitude found in patients not selecting toric contact lenses or intraocular lenses.

## Methods

### Subjects

A total of 54 subjects (mean [SD] age: 56 [18] yr) were recruited for the VA task, including 33 normally sighted controls (mean [SD] age: 48 [17] yr) and 21 patients (mean [SD] age: 68 [11] years) with anterior and/or posterior segment ocular pathologies. The total number of subjects or patients was decreased in analyses for which a patient did not complete the other parts of the study or age-balancing was needed to compare patients to controls. A limited review of clinic records from the Indiana University School of Optometry identified patients with diagnoses concerning anterior segment, posterior segment, or both. OCT and scanning laser ophthalmoscopy images were acquired using the Spectralis OCT II (Heidelberg Engineering, Heidelberg, Germany) and graded by an ophthalmologist to identify posterior segment abnormalities and provide an update beyond chart information. Slit lamp examination was used to assess anterior segment conditions. Older controls were permitted to have retinal changes consistent with normative aging, such as a few small drusen or epiretinal membrane no greater than Stage 2. Chart information plus these tests classified the patients as differing from older controls as follows: 8 with posterior segment changes, 3 with anterior segment changes, and 6 with both anterior and posterior segment changes, including macular pucker, epiretinal membrane, drusen, cystoid macular edema, dry eye disease, posterior capsular opacification, and cataract (Table 1). The numbers of subjects in some analyses of induced aberration conditions are reduced, due to missing VA data. Similarly, when subgroup analyses were performed the sample size decreased, e.g., age-balanced groups or subjects with posterior conditions only. When a total number of 30 controls was used, there were 13 males and 17 females. When a total number of 17 patients was used, there were 6 males and 11 females.

**Table 1:** Distribution of ocular conditions across subjects with posterior and/or anterior segment conditions.

	Posterior + Anterior conditions	Posterior conditions only	Anterior conditions only
**No. of Subjects**	6	8	3
**Macular Edema**	1	2	–
**CMT > 301 microns**	1	3	1
**DRIL**	1	1	–
**Macular Pucker/Retinal Folds/ERM**	3	2	–
**Microaneurysms**	6	2	–
**Diabetes**	2	3	–
**AMD**	1	1	–
**Drusen**	4	3	–
**Glaucoma**	1	2	–
**Cataract**	2	–	2
**PCO**	2	–	–
**Dry Eye**	3	–	1
**High Myope/LASIK**	1	–	2

Abbreviations: CMT = Central macular thickness, DRIL = Disorganization of inner retinal layers, ERM = Epiretinal membrane, AMD = Age related macular degeneration, PCO = Posterior capsular opacification, LASIK = Laser-assisted in situ keratomileusis.

Clinical VA was measured under habitual correction using a standardized electronic Early Treatment Diabetic Retinopathy Study (ETDRS) computerized vision testing system (SmartSystem 2, M&S Technologies, Niles, IL, USA). Controls had VA better than 20/25, while patients had VA better than 20/40. All subjects were consented and tested in a manner approved by the Indiana University Institutional Review Board, which adhered to the Declaration of Helsinki. Informed consent was obtained from the subjects after an explanation of the nature and possible consequences of the study. The consent was written and witnessed by a member of the study team on the IRB protocol. The start date of the recruitment was June 1, 2022, and the end of the recruitment was May 23, 2024.

Wavefront aberrations of the eye were collected as described previously [13] using a commercial system: Pentacam AXL Wave (Oculus, Wetzlar, Germany). For each eye, three consecutive measurements were acquired and averaged to obtain individual Zernike coefficients, as well as the root mean square (RMS) error of lower-order (RMSLO) and higher-order aberrations (RMSHO). Measurements used the subject’s natural pupil, with aberration data for the 3 mm pupil diameter used for analysis, while data for the 5 mm pupil provided documentation of significantly larger aberrations, as previously reported for 37 control subjects [13]. Wavefront measurements were successfully obtained in 49 of the 54 subjects recruited, with anterior segment issues such as dry eye being the cause of failure to acquire data. Subjects with missing wavefront data were excluded from the wavefront analysis. Note that the patients recruited specifically included a subset with diagnosed anterior segment issues. Two additional subjects were removed from the sample because they could not follow instructions, giving a total of 47 subjects. Individual subject information and details about the refractive status were provided in S1 Text.

#### VA task.

The VA measurements were acquired using the PVT (Aeon Imaging, LLC, Bloomington, IN, USA) that was designed to project the visual stimulus using Maxwellian view and a 3 mm pupil, as previously described [27]. All 47 subjects completed the task and provided good VA measurements. The VA task consisted of four alternative forced choice trials [27,46–49] with a single black tumbling E on an approximately 5 deg white background, to avoid crowding and reduce glare. VA stimuli were presented on a high-resolution display achieving a visual angle of approximately 0.25 arcmin per pixel on the retina. The letter sizes varied between a minimum angle of resolution (MAR) of 0.5 and 10 arcmin and changed in 0.1 logMAR steps. At each letter size, 8 consecutive trials were presented. When there were > 70% correct responses at a given size, the letter size was reduced to a smaller size; otherwise, the letter size was increased. Subjects provided the responses using a keypad. Continuous viewing of the E was allowed to mimic the standard clinical VA tests. This method provided a full psychometric function.

The PVT setup included a Badal optometer that provided continuously adjustable defocus correction and a piezoceramic deformable mirror (DM) (AOK8, DM and HS kit, Thorlabs, Newton, NJ, USA) positioned at the pupil plane to induce specific wavefront aberrations. Before the start of the VA task, the DM was set to the flat position, the subject fixated on a letter E (20/60), and the Badal optometer was adjusted to achieve the best subjective focus. In our previous study [27], the subjects had minimal astigmatism, and therefore we could not analyze the effects of astigmatism with Maxwellian view and a 3 mm pupil. For this study, the astigmatism obtained from the autorefractor ranged from −0.21 to −2.63 D for controls, and 0 to −3.21 D for patients. We analyzed separately the VA for the 10 controls with astigmatism ≤ 0.5 D and the 20 with > 0.5 D. The lack of correction for astigmatism allowed us to investigate the potential for a significantly lower cost and less complex instrument, i.e., one that allowed for spherical correction alone and no additional aberrations. This provided a test of whether VA measured with Maxwellian view and a 3 mm pupil led to the expected decrease in VA due to astigmatism or instead minimized the impact of astigmatism sufficiently to detect no difference between groups or an association from regression analyses.

In this paper we report not only on the zero induced aberrations, which may include inherent aberrations, but also induced aberrations as large as or larger than aberrations encountered with a 5 mm or larger pupil [27]. The goal was to simulate the effects on the VA of large pupil size or more extreme aberrations. The controls were tested with the full protocol [27] using previously calibrated aberrations: 0 induced aberration: spherical aberrations −0.32, −.023, + 0.27, + 0.39 microns; and X coma of +0.63, −0.51, and Y coma of +0.49, −0.59 microns. As this proved challenging for the patients with the more severe changes to their posterior and/or anterior segments, we did not test all patients for these conditions, decreasing the sample size for induced aberrations. Individual subject VA data are provided in S2 and S3 Text.

### Statistical analysis

The VA data were fit with a cumulative Gaussian function using the least root mean squared error minimization and custom MATLAB software (MathWorks, Natick, MA, USA) as previously [27,31,50–52]. The letter size with 50% correct responses was considered the mean VA or the 50% threshold VA. The 50% threshold VA size was obtained from the cumulative Gaussian function used to fit the VA data. This method provided the SD for each subject, directly from the fit functions. The variability in VA responses was quantified as the coefficient of variation (CV), calculated as the SD normalized by the mean VA. The psychometric function fits were performed using letter sizes in MAR, and the VA metrics were converted to logMAR.

To investigate the association between the mean VA and the variables including age, astigmatism, and RMSHO, we performed a general linear model (GLM) analysis. For controls the sample size was 30, and resultant degrees of freedom were 23. For patients the sample size was 17, and resultant degrees of freedom were 10. In addition to the full model incorporating all three variables and their interactions, separate GLM and Pearson product moment correlation coefficient analyses were conducted for each variable individually to examine their potential maximum effects on VA. For analyzing VA changes with astigmatism, we removed one outlying point and refitted the data to see if we could improve the fit of the data; however, the p-values in the text and tables represent the full data set. ANOVA was also performed for the SD and CV. We performed two-sample student t-tests to compare the controls and patients when the variances were equal, and the Satterthwaite t-test when the variances were unequal. In addition, paired t-tests were performed when two values of the same subject were compared, e.g., for astigmatism and for spherical aberration. Repeated measures ANOVA was performed with mean VA as the dependent variable and disease status (control vs patient) and induced aberration condition with repeated measures as independent variables. For analyses in which controls and patients were in separate tests, or when age was a covariate, the full sample size for controls was used. However, when comparing patients to controls, the control group was subsampled to achieve an age-balanced group with the degrees of freedom adjusted for the diminished sample size, as described in Results. For instance, the VA analysis had a mean age of 61 [13] yr for age-balanced controls compared to 68 [11] yr for patients. Correlation among variables was assessed using linear regression fits, and the R scores were converted to p-values using a web-based tool (socscistatistics.com). The significance level of 0.05 for p values was considered for all statistical tests and Bonferroni correction was applied whenever it was required. The statistical analyses were performed using on demand SAS Studio (Cary, NC, USA).

## Results

**Fig 1:**
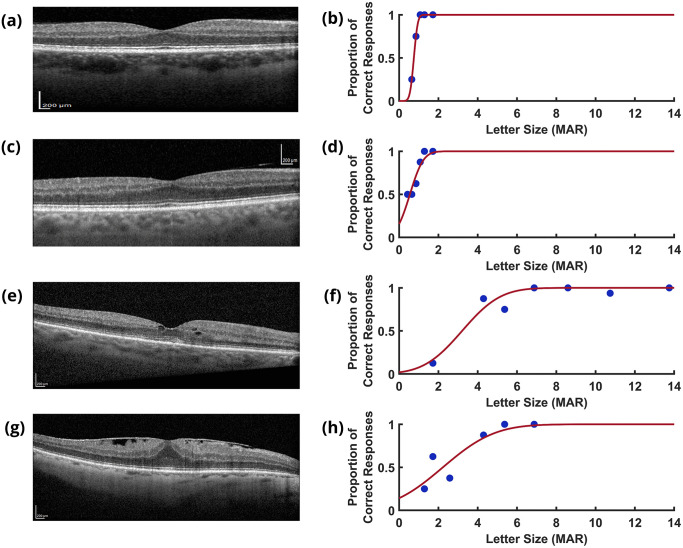
Foveal B-scan and VA data (blue circles) and the psychometric function fit with a cumulative Gaussian function (red line) for selected subjects. **(a)** B-scan of a 29 yr old female control with a clear foveal pit with photoreceptors having long outer segments in the fovea and well-organized retinal layers with CMT = 301 microns; (b) data and fit showing excellent VA (0.75 MAR) and fit with a steep slope that indicates a small SD (0.16 MAR); **(c)** B-scan of a 75 yr old male control with a less prominent foveal pit but photoreceptor outer segments still longer in the foveal region and well-organized retinal layers with CMT = 320 microns; (d) data and fit showing excellent VA and fit with an excellent mean VA (0.55 MAR) and a shallower slope indicating a somewhat larger SD (0.54 MAR); **(e)** B-scan of a 75 yr old female diabetic subject showing cystoid macular edema, DRIL, changes to photoreceptor outer segments, and microaneurysms with CMT = 331 microns; (f) data and fit showing worse consistency of the data and a larger mean VA (3.21 MAR). The shallower slope indicated a larger SD (1.53 MAR); **(g)** B-scan of a 70 yr old male subject with macular edema, retinal folds, and macular pucker. The foveal region is unusually thick and lacks a foveal pit, with CMT = 495 microns, and the foveal photoreceptor outer segments are short. **(h)** Data and fit showing worse consistency of the data and a larger mean VA (2.23 MAR). The shallower slope indicated a larger SD (2.08 MAR).

The VA data were well-fit by the cumulative Gaussian function for most subjects including both patients and controls (Fig 1). Mean VA and SD were quite good for most subjects, especially for those with astigmatism ≤ 0.5 D (Tables 2 and 3).

**Table 2:** Numbers of subjects for controls, and corresponding averages for Age, Astigmatism, Sphere, RMSHO, Mean VA, SD of VA, and CMT.

	Overall Control	Astigmatism less than or equal to 0.5 D	Astigmatism greater than 0.5 D
**N**	30	10	20
**Age Mean (SD)(Years)**	46 (17)	34 (7.6)	53 (17)
**Sphere Mean (SD)(Diopters)**	−1.1 (2.03)	−1.3 (1.67)	−0.98 (2.21)
**Astigmatism Mean (SD) (Diopters)**	−0.91 (0.68)	−0.34 (0.10)	−1.2 (0.67)
**RMSHO Mean (SD) (microns)**	0.076 (0.051)	0.050 (0.012)	0.088 (0.058)
**50% Threshold VAMean (SD) in MAR[logMAR]**	0.99 (0.43)[−0.0046 (−0.37)]	0.75 (0.21) [-0.12 (-0.68)]	1.1 (0.46) [0.045 (-0.34)]
**SD of VAMean (SD) in MAR [logMAR]**	0.50 (0.44)[−0.30 (−0.36)]	0.29 (0.27) [-0.54 (-0.58)]	0.61 (0.47) [-0.21 (-0.32)]
**CMT Mean (SD) (microns)**	273 (22.2)	269 (21.3)	275 (22.9)

Abbreviations: RMSHO = Root Mean Squared Error, CMT = Central macular thickness.

**Table 3:** Numbers of subjects for patients, and corresponding averages for Age, Astigmatism, Sphere, RMSHO, Mean VA, SD of VA, and CMT.

	Total Patients	Posterior only	Posterior + Anterior	Anterior
**N**	17	8	6	3
**Age Mean (SD)(Years)**	68 (13)	66 (17)	73 (6.6)	62 (11)
**Sphere Mean (SD)** **(Diopters)**	−0.74 (3.09)	0.40 (1.88)	−1.2 (3.51)	−2.9 (4.51)
**Astigmatism Mean (SD) (Diopters)**	−1.4 (0.76)	−1.3 (0.56)	−1.5 (1.08)	−1.5 (0.70)
**RMSHO Mean (SD) (microns)**	0.13 (−0.052)	0.11 (0.040)	0.16 (0.067)	0.14 (0.041)
**50% Threshold VAMean (SD) in MAR** **[logMAR]**	1.5 (0.59)[0.16 (−0.23)]	1.5 (0.80)[0.18 (−0.096)]	1.5 (0.39)[0.18 (−0.41)]	1.2 (0.24)[0.093 (−0.62)]
**SD of VAMean (SD) in MAR** **[logMAR]**	0.54 (0.55)[−0.26 (−0.26)]	0.52 (0.49)[−0.28 (−0.31)]	0.72 (0.72)[−0.14 (0.14)]	0.25 (0.24) [-0.60 (-0.62)]
**CMT Mean (SD)** **(microns)**	298 (56.7)	295 (31.0)	312 (90.7)	279 (26.1)

Abbreviations: RMSHO = Root Mean Squared Error, CMT = Central macular thickness.

**Fig 2:**
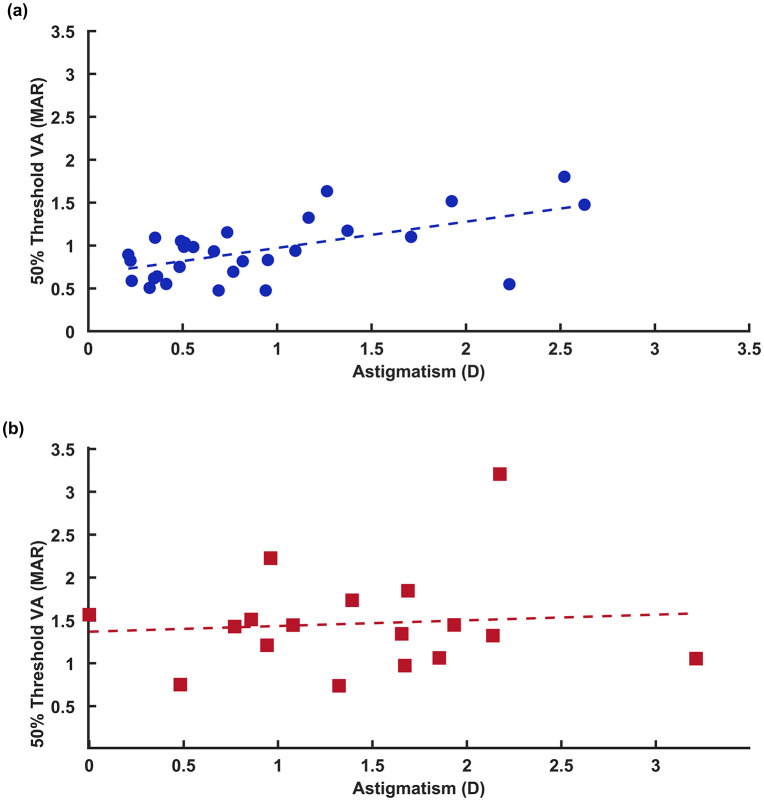
Mean VA of individual subjects as a function of astigmatism in diopters (negative cylinder). (a) controls shown as blue with one outlying point removed, and (b) patients shown as red squares. The dashed lines indicate the linear regression fits for subjects, which were not significant for the Pearson product moment fits. However, the GLM fit was significant for the controls (*p* = 0.011).

The results of the general linear model revealed that for controls there was a trend for the association between VA and astigmatism (*p* = 0.052), and therefore this was investigated as a single variable (Table 4). The separate GLM analysis including only astigmatism as a predictor, without correcting age or RMSHO, indicated a significant association with VA for controls (*p* = 0.011) (Fig 2). While the GLM fit was significant for the controls, the Pearson product moment correlation was not significant, due to a different computation of significance from an identical R^2^ value. None of the following continuous variables had trends indicating associations: age, RMSHO, and interactions between these variables and astigmatism. Further, in a post hoc test of sex as a biological variable, there was no significant difference in VA measurements for males vs females: controls with astigmatism ≤ 0.5 D (*p* = 0.067), controls with astigmatism > 0.5 D (*p* = 0.21), and combined control group (*p* = 0.40).

**Table 4:** Results from the general linear model for the mean VA.

	Controls		Patients	
	F Value (DF = 1,23)	P Value	F value (DF = 1, 9)	P Value
**Age**	0.65	0.43	0.030	0.87
**RMSHO**	0.05	0.83	1.7	0.22
**Astigmatism**	4.2	0.052	2.1	0.18
**Age x Astigmatism**	1.4	0.25	2.6	0.14
**RMSHO x Astigmatism**	0.010	0.91	0.050	0.82
**Age x RMSHO**	0.040	0.84	1.4	0.27

**Fig 3:**
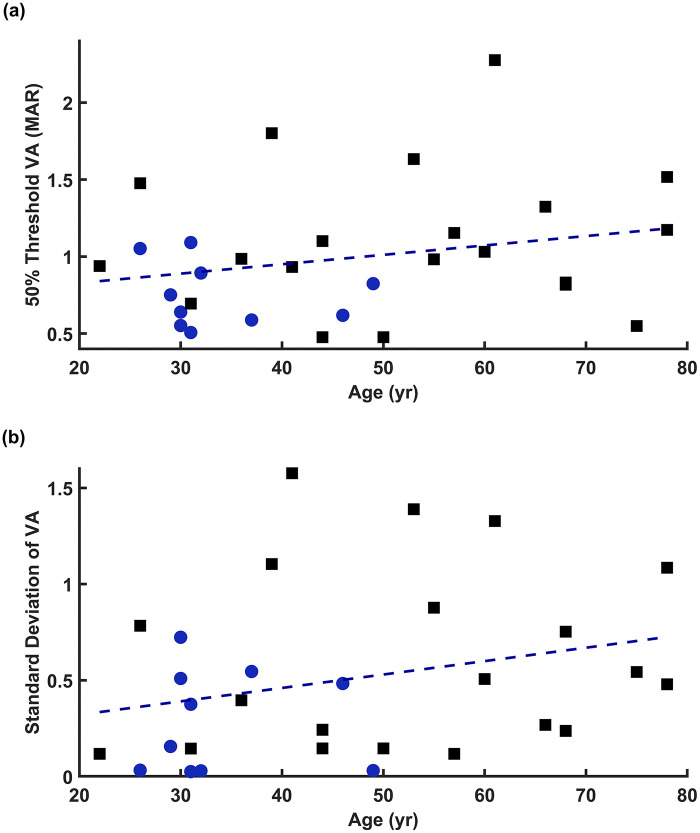
VA metrics of the individual control subjects plotted as a function of age. (a) 50% threshold VA and (b) standard deviation of psychometric function. The blue dots represent the controls with astigmatism less than 0.50 D, and the black squares represent controls with astigmatism larger than 0.5 D. The blue dashed line indicates the linear regression fit for the overall control group, which was not statistically significant.

When examining one variable at a time, the mean VA (50% threshold) did not change significantly with age for either the 10 controls with minimal astigmatism (≤ 0.5 D) (R^2^ = 0.036, *p* = 0.93) or the 20 controls with astigmatism > 0.5 D (R^2^ = 0.0038, *p =* 0.99) (Fig 3a). In agreement with the typical clinical finding, the mean VA was better for the controls with minimal astigmatism compared with astigmatism > 0.5 D (MAR Mean (SD): 0.75 (0.21) vs 1.1 (0.46), logMAR: −0.12 (−0.68) vs 0.045 (−0.34), t (28) = 2.3, *p* = 0.027).

Unlike the mean VA, the variability measures for VA, and SD and CV of VA, were not significantly different among controls with minimal astigmatism compared to controls with astigmatism> 0.5 D (SD: t (28) = 2.0, *p* = 0.057; CV: t (28) = 0.36, *p* = 0.72). Similar to mean VA, the variability measures of VA as a function of age, i.e., the SD and CV, also did not change significantly for the controls with minimal astigmatism (SD: R^2^ = 0.00042, *p* = 0.99; CV: R^2^ = 0.00022, *p* = 0.99). For the controls with astigmatism > 0.5 D, the SD and CV of VA also did not change as a function of age (SD: R^2^ = 0.013, *p =* 0.97; CV: R^2^ = 0.035, *p* = 0.88) (Fig 3b).

**Fig 4:**
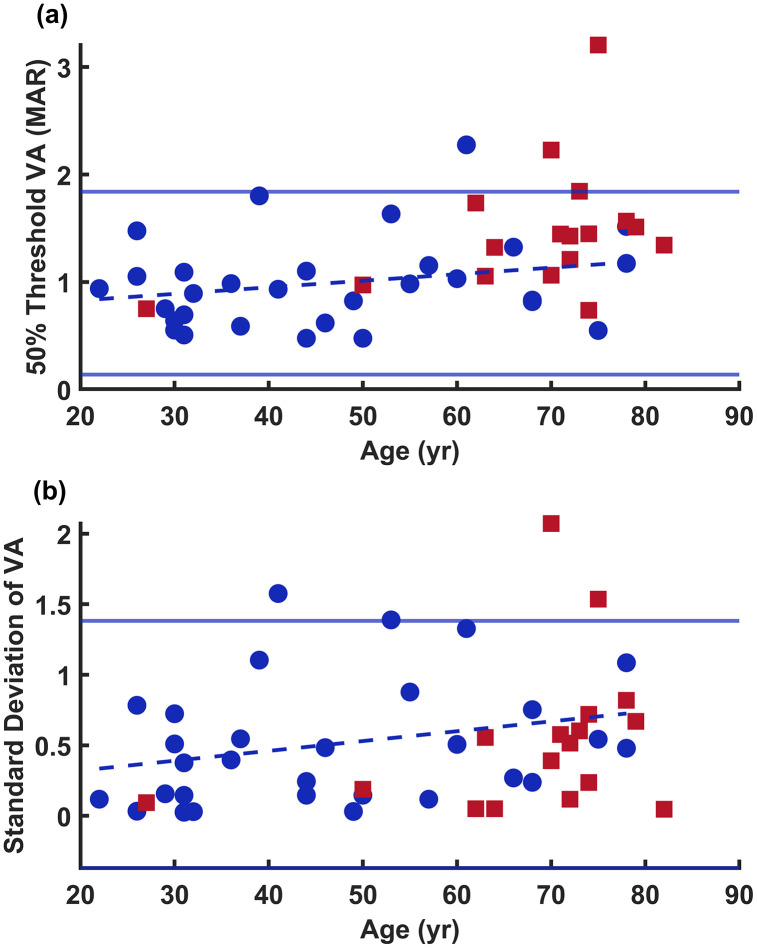
VA metrics of individual subjects as a function of age. (a) 50% Threshold VA and (b) standard deviation of VA. The blue dots represent the controls, and the red squares represent the patients. The blue dashed lines indicate the linear regression fits for controls. The solid blue line represents the confidence limits (computed as mean + /- 2*SD) for the controls. The lower limits were less than zero and not shown in the figure.

For patients, the GLM analysis indicated that there was no significant association between the mean VA and any of the following continuous variables: age, astigmatism, RMSHO, and their interactions (Table 4) (Fig 4a). Unlike the controls, the patients did not show an effect of astigmatism on VA in a single variable regression analysis (*p =* 0.74) (Fig 2b). For subgroups of patients, no significant change in mean VA was observed with age for either the 6 patients with both anterior and posterior conditions (*p* = 0.97) or the 8 patients with posterior conditions alone (*p* = 0.72) The mean VA was not significantly different among patients with anterior and posterior segments conditions and patients with posterior segment conditions alone (MAR: 1.5 (0.39) vs 1.5 (0.80); logMAR 0.18 (−0.41) vs 0.18 (−0.096), t (12) = 0.019, *p* = 0.98).

Similar to mean VA, the variability measures for individual subject VAs, i.e., SD and CV, also did not change significantly with age for the patients with anterior and posterior segment conditions (Fig 4b). The regression indicated that SD = −0.037 * age + 3.43, R^2^ = 0.12, *p* = 0.82, and CV = −0.026 * age + 2.34, R^2^ = 0.30, *p* = 0.56. For patients with posterior segment conditions alone, the SD = 0.015 * age −0.46, R^2^ = 0.25, *p =* 0.54, and CV = 0.0081 * age −0.18, R^2^ = 0.20, *p* = 0.62. The SD and CV of VA were not significantly different among patients with anterior and posterior segments conditions compared to patients with posterior segment conditions alone (SD: t (12) = −0.61, *p* = 0.55; CV: t (12) = −0.45, *p* = 0.66).

The two patients with extremely high SD values had significant retinal pathologies. One had cystoid macular edema, microaneurysms, and DRIL (Fig 1e). The other had retinal folds, drusen and macular pucker (Fig 1g).

In agreement with the general linear model results above, the analysis of single variables showed that the mean VA (50% threshold) did not change significantly with age for either controls (*p* = 0.90) or patients (*p =* 0.55) (Fig 4). The mean VA of individual subjects was significantly worse for patients compared to the full sample of controls (MAR: 1.5 (0.59) vs 1.0 (0.44), logMAR: 0.16 (−0.23) vs 0.00 (−0.36); t(45) = −3.0, *p =* 0.0047). However, the mean VA of individual subjects was not significantly different for patients compared to the age-balanced control group (t(28) = 1.3, *p* = 0.18 for age) (MAR: 1.5 (0.59) vs 1.1 (0.49); logMAR: 0.16 (−0.23) vs 0.048 (−0.31); t(28) = −1.7, *p* = 0.090). The variability was not worse for patients compared to the age-balanced control group (SD of individual VA: 0.66 (0.44) vs 0.54 (0.55); logMAR: −0.26 (−0.26) vs −0.22 (−0.35); t (28) = 0.34, *p* = 0.74).

### Higher order wavefront aberrations

#### RMSHO.

**Fig 5:**
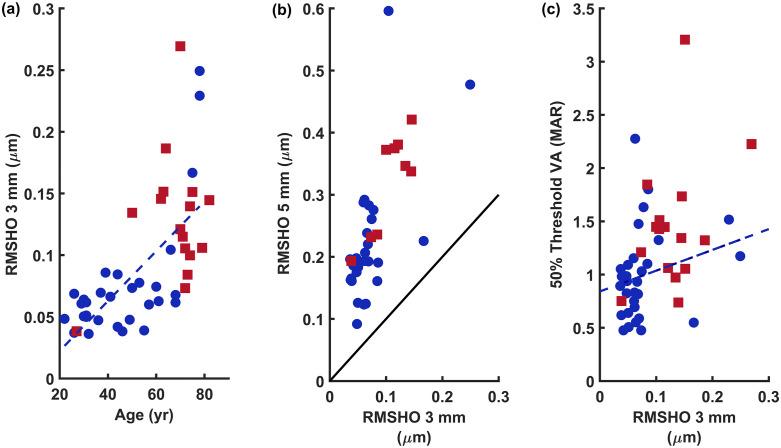
Relation of RMSHO with age, pupil size, and VA. **(a)** RMSHO for a 3 mm pupil as a function of age, **(b)** RMSHO for a 5 mm pupil as a function of RMSHO for a 3 mm pupil. The black solid line has a slope of 1; all points lie above the line indicated larger RMSHO for the 5 mm pupil. **(c)** The mean VA (50% threshold VA) as a function of RMSHO for a 3 mm pupil. The blue dots represent the controls, and the red squares represent the patients. The blue dashed lines indicate the linear regression fits for the controls.

The RMSHO aberrations were significantly reduced in controls for the 3 mm pupil compared to the 5 mm pupil (mean difference: −0.058 (0.071) microns, t (27) = −4.3, *p* < 0.001, Fig 5). The data point for each subject was less for a 3 mm pupil than a 5 mm pupil (Fig 5b). This significant reduction of RMSHO for 3 compared to 5 mm pupil was also found for patients (mean difference: −0.22 (0.053) microns, t (9) = −12, *p* < 0.001). Furthermore, patients had significantly larger RMSHO values compared to the whole control group (mean (SD) RMSHO 3 mm: 0.13 (0.052) vs 0.076 (0.051) microns, (t (44) = −3.35, *p* = 0.0016). However, RMSHO did not differ significantly 0.10 (0.068) vs 0.13 (0.052) microns, (t (27) = −1.2, *p* = 0.24) when the age of the control group was balanced to that of the patients (t (28) = 1.3, *p* = 0.18 for age).

Among controls, RMSHO significantly increased with age (RMSHO = 0.0020 * age – 0.018, R^2^ = 0.45, *p* = 0.014). In contrast, there was no significant change in RMSHO with age for patients (*p* = 0.76) (Fig 5a), who had a more limited range except for the youngest patient. However, the mean VA did not significantly change with RMSHO for a 3 mm pupil either for controls (R^2^ = 0.055, *p* = 0.77) or patients (R^2^ = 0.15, *p* = 0.55) (Fig 5c). Similarly, SD did not change significantly with RMSHO (3 mm) for both controls (R^2^ = 0.069, *p* = 0.72) and patients (R^2^ = 0.36, *p* = 0.16).

#### Spherical aberrations.

**Fig 6:**
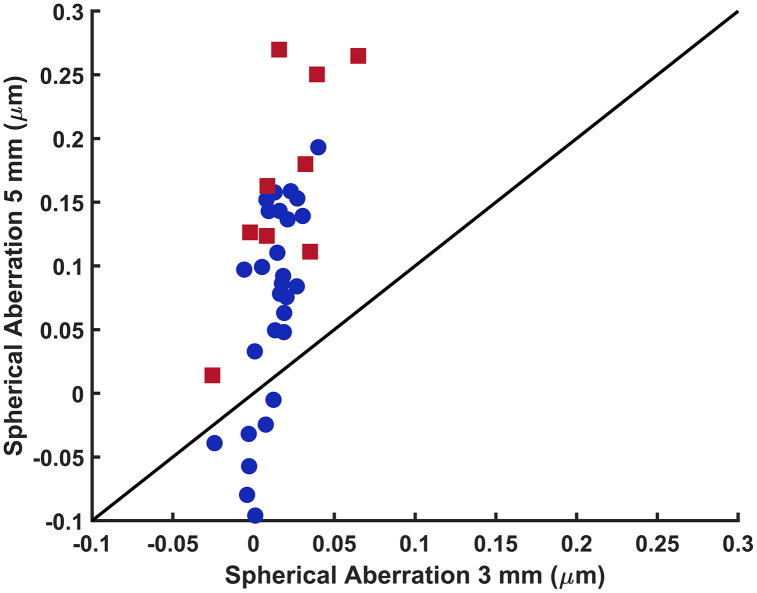
Spherical aberration for a 3 mm pupil vs. a 5 mm pupil. **(a)** Control subjects. **(b)** Patients. The blue dots represent the controls, and the red squares represent the patients. The solid black line indicates equality.

As expected, there was significantly less spherical aberration for the 3 mm pupil than for the 5 mm pupil for controls, as previously reported [13], and similarly for patients (Fig 6). To compare the means of the values of spherical aberration for 3 vs. 5 mm pupils, absolute values were used because there were both positive and negative values. The spherical aberration for the 3 mm pupil was mean (SD): 0.013 (0.014) microns vs the 5 mm: 0.070 (0.080) microns; paired t-test: t (27) = −4.3, *p* < 0.001 for controls. For patients, the spherical aberration values were smaller for the 3 mm pupil: 0.024 (0.025) microns vs the 5 mm: 0.17 (0.085) microns; paired t-test: t (8) = −12, *p* < 0.001.

**Fig 7:**
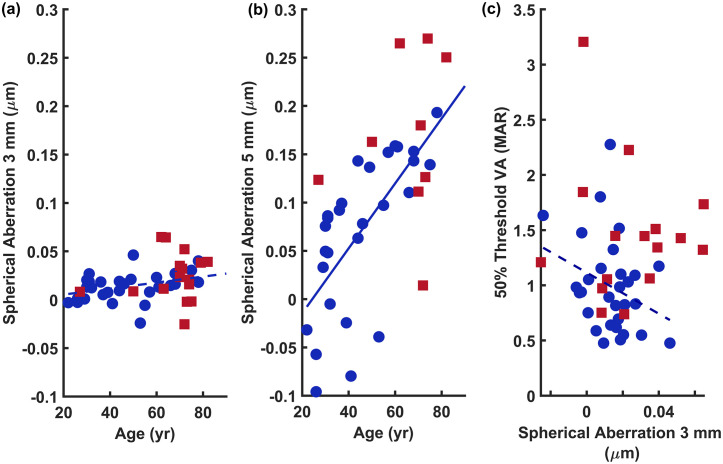
Relation of Spherical Aberrations with age and VA. Spherical aberration as a function of age for (a) a 3 mm pupil diameter and (b) a 5 mm pupil diameter. **(c)** The mean VA (50% Threshold) as a function of spherical aberration for a 3 mm pupil. The blue dots represent the controls, and the red squares represent the patients. The blue dashed and solid lines indicate the linear regression fits for controls, with dashed lines indicating associations that are not significant.

For the 3 mm pupil there was no significant change in spherical aberration with age either for controls (R^2^ = 0.14, *p* = 0.48) or patients (R^2^ = 0.012, *p* *=* 0.96) (Fig 7). For the 5 mm pupil, there was a significant positive correlation between spherical aberration and age for controls (spherical aberration = 0.0034 * age – 0.082, R^2^ = 0.47, *p* = 0.0091) (Fig 7). However, no significant correlation was found for patients (R^2^ = 0.037, *p* = 0.89), although age varied over a smaller range that included mostly older individuals.

The mean VA did not change significantly with inherent spherical aberrations for a 3 mm pupil for either the controls (MAR = −9.3 * spherical aberration + 1.2, R^2^ = 0.097, *p =* 0.61) or patients (MAR = −1.9 * spherical aberration + 1.5, R^2^ = 0.0062, *p* = 0.98) (Fig 7). Similarly, the SD of the VA measurements did not change significantly with inherent spherical aberrations either for controls (R^2^ = 0.11, *p* = 0.57) or patients (R^2^ = 0.0038, *p* = 0.89).

### Central macular thickness

**Fig 8:**
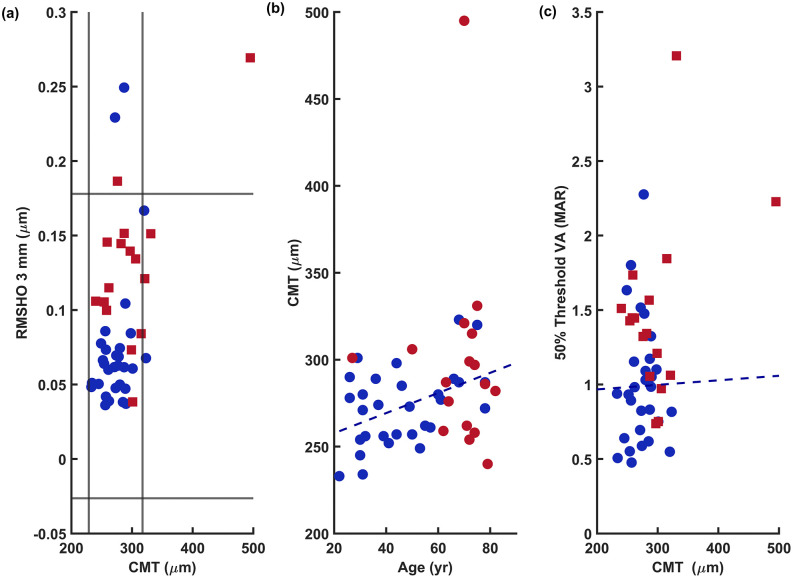
Relation of CMT with RMSHO, age, and VA. **(a)** RMSHO (microns) for a 3 mm pupil diameter as a function of CMT (microns). The black lines indicate the confidence limits (mean + /- 2*SD), **(b)** CMT as function of age, and (c) mean VA (50% threshold VA) as a function of CMT. The blue dots represent the controls, and the red squares represent the patients. The blue dashed lines represent the linear regression fits for controls, which did not reach statistical significance.

CMT ranged from 233 to 323 microns for controls and 240–495 microns for patients (Fig 8). Only five subjects, 3 of 17 patients and 2 of 30 controls, had CMT values outside the 95% confidence interval of the present controls, indicating a sample that does not contain a high proportion of severely exudative eyes. The CMT was not significantly different between the age-balanced controls and patients (t (28) = −0.97, *p* = 0.34). The means (SD) for age-balanced controls and patients were 281 (23) vs 298 (57) microns. The RMSHO did not significantly change with CMT for either controls (R^2^ = 0.079, *p* = 0.68) or patients (R^2^ = 0.40, *p* = 0.13) (Fig 8). Among all the subjects, only one subject with macular edema and macular pucker had both RMSHO and CMT larger than the mean + /- 2*SD of the controls. There was no significant change in CMT with age for either controls (R^2^ = 0.20, *p* = 0.30) or patients (R^2^ = 0.0030, *p* = 0.99).

The mean VA did not change significantly with CMT for either controls (R^2^ = 0.00025, *p* = 0.99) or patients (R^2^ = 0.14, *p* = 0.60). Similar to the mean VA, the SDs of VA measurements for individual subjects did not change significantly with CMT for the controls (p = 0.97). However, for patients the SD of VA measurements for individual subjects increased significantly with CMT (R^2^ = 0.54, *p* = 0.025).

### Induced aberrations

**Fig 9:**
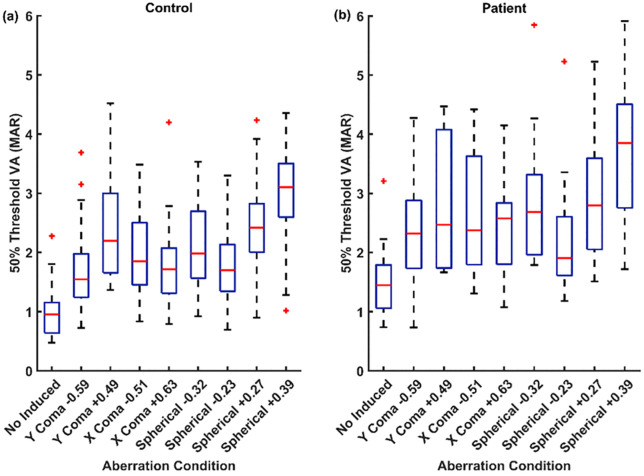
The pooled mean VA (50% threshold) plotted for each induced aberration condition. The pooled mean VA (50% threshold) plotted for each induced aberration condition for (a) all controls and (b) patients.

There was a significant difference in the mean VA among induced aberration conditions (Fig 9), for the full control sample (F (8,29) = 24, *p* < 0.001) and the patient sample (F (8,11) = 6.1, *p* < 0.001), as determined by repeated measures ANOVA. The paired t-tests showed that VA was better for no induced aberrations compared to any of the induced aberration conditions, with *p* values < 0.001 for the full sample of controls, and *p*-values ranging from 0.0051 to < 0.001 for patients, all less than the Bonferroni corrected p-value: < 0.0062.

In order to compare the VA of patients to controls, we again formed an age-balanced control group, with no statistically significant difference in age (t(17) = 1.7, *p* = 0.92). The mean VA was significantly better for the age-balanced control group compared to the patient group only for induced negative coma conditions, i.e., Y coma −0.59 µm (t(17) = −3.2, *p* = 0.0055), X coma −0.51 µm (t(17) = −2.6, *p* = 0.029). No significant difference was observed in mean VA for the rest of the induced aberration conditions between the age-balanced control group and patients.

**Fig 10:**
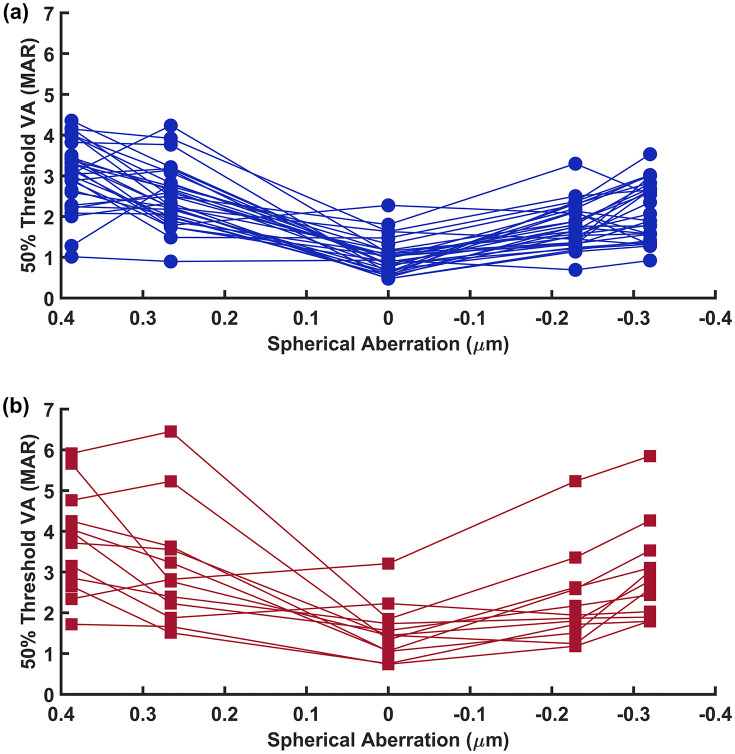
Mean VA (50% threshold) as a function of spherical aberrations. The VA worsened with increasing amounts of induced spherical aberrations for (a) controls (blue dots) and (b) patients (red squares). For most subjects the slope of the VA as a function of spherical aberrations was well-fit by two linear functions, one for positive and another for negative with zero as the best VA.

**Fig 11:**
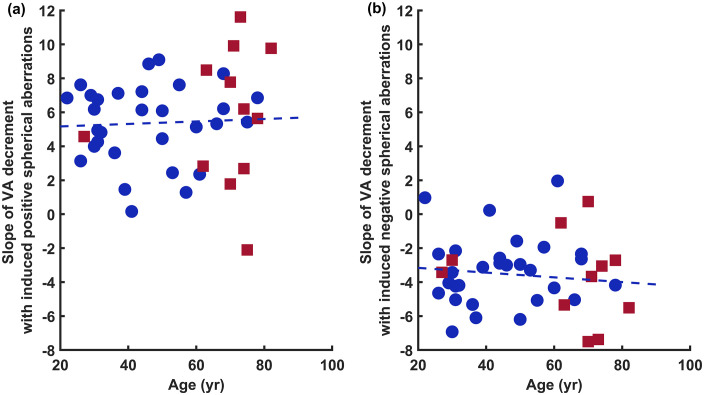
Slopes of VA decrement with induced spherical aberration (MAR/micron) as a function of age. VA decrement for (a) induced positive spherical aberrations and (b) induced negative spherical aberrations. Blue dots represent controls, and red squares represent the patients. The blue dashed line represents the linear regression fit for controls.

For most subjects there was a linear decline in VA with increasing amounts of spherical aberrations, as found previously (Fig 10) [27]. This finding indicates that the effect on VA of spherical aberrations can be analyzed using the slope of VA decrement vs amount of aberration, without squared or other higher order terms. For each subject, the slope for regression fits for mean VA (MAR/micron) was computed as a function of spherical aberration level. The individual subject’s slope of VA decrement with induced positive spherical aberration level did not change significantly with age for either the controls (R^2^ = 0.0025, *p* = 0.99) or the patients (R^2^ = 0.014, *p* = 0.99) (Fig 11). Similarly, there was no significant change in slope for negative spherical aberration levels with age for controls (R^2^ = 0.0096, *p* = 0.95) or patients (R^2^ = 0.064, *p* = 0.84).

The slope of VA decrement across induced spherical aberration level was significantly steeper for positive spherical aberrations compared to negative spherical aberrations. In control subjects, the slope of VA decrement for positive induced spherical aberrations was significantly greater than negative ones with a mean difference in slopes = 8.9 (3.5) (paired t-test: t (29) = 04.16, *p* < 0.001) (Fig 11). In patients as well, the slope of VA change for positive induced spherical aberrations was significantly greater with a mean difference in slopes = 9.9 (5.5) (paired t-test: t (11) = 6.3, *p* < 0.001).

The inherent spherical aberrations in our controls and patients for the 3 mm pupil condition were minimal (Fig 6) compared to the induced aberrations. Similarly, the slope of the decrease in VA with spherical aberration was roughly linear for both positive and negative spherical aberrations, so that an offset does not influence the slope. Thus, inherent spherical aberrations did not contribute significantly to the difference between the VA for the large induced positive vs. negative spherical aberrations.

We further investigated hypotheses that could explain why the induced positive spherical aberrations worsened VA to a greater extent than negative spherical aberrations. First, we computed the total system wavefront aberrations for the most extreme positive spherical aberration condition. We used the Zernike values that were calibrated for each value used of the induced aberrations [27]. The moderate cost deformable mirror differed somewhat for calibrated vs. nominal values, as measured with the independent illumination source and wavefront sensor described previously [27]. For the greatest positive spherical aberration, the value was 0.39 microns of error while for the negative the value was only 0.32 microns. Further, there were errors for other Zernike terms. However, the total RMS error in microns for the greatest amplitude negative spherical aberration tested was larger, not smaller, than the corresponding positive spherical aberration value tested: 0.30 vs. 0.28 microns. That is, there was less induced total aberration for the positive spherical aberration condition, which does not explain our findings. To determine whether the slight difference in positive vs. negative aberration had an effect on VA, we recomputed the amount of VA decrement for the same amplitude of positive and negative aberration, i.e., scaled all the VA values for the positive spherical aberration. Among controls, the mean difference was 0.61 (0.94) MAR, with VA for the positive condition significantly larger: 2.7 (0.64) MAR, paired t-test t(29) = 3.6, *p* = 0.0012. However, no significant difference was observed among patients, paired t-test t(11) = 1.4, *p* = 0.17.

We additionally investigated whether accommodation in the negative induced spherical aberration conditions could overcome defocus that could not be compensated for in the positive spherical aberration conditions. More compensation due to accommodation could be the case for younger, but not older, subjects. For the greatest amplitude positive and negative spherical aberrations, we performed a regression against age for the difference between the VA for scaled positive aberration and the VA for the negative aberration. There was no effect of age: VA = 0.0012 * age + 0.59, R2 = 0.00043. This is supported by the findings of no trends in VA with age for either controls or patients for induced positive aberrations or negative aberrations. The lack of an effect of age also argues against smaller pupil sizes for negative spherical aberrations and younger subjects, which is credited for improving VA for near vision in the presence of negative spherical aberrations. There were also no significant associations with any refractive variables. The results are consistent with an optical explanation, in which the higher spatial frequency information from the periphery of the pupil is decreased more for the positive than for the negative spherical aberration conditions [53,54].

Tables 5 and 6 present a summary of the main findings of this study, showing the effect of astigmatism on VA but not other inherent aberrations. However, large induced aberrations, similar to the magnitude found for 5 mm pupils, do lead to consistent decrements in VA.

**Table 5:** Summary statistics indicating statistical significance for inherent aberrations.

Statistical Comparisons	Controls	Patients	Controls vs Patients	Age Balanced Controls Vs Patients
**Multivariate Analysis for VA**				
Age	–	–	NA	NA
RMSHO	–	–	NA	NA
Astigmatism	*	–	NA	NA
Age x Astigmatism	–	–	NA	NA
RMSHO x Astigmatism	–	–	NA	NA
Age x RMSHO	–	–	NA	NA
**Univariate Analysis**				
Association between VA and Astigmatism	**	–	NA	NA
Association between Mean VA vs Age	–	–	NA	NA
Astigmatism ≤ 0.5	–	NA	NA	NA
Astigmatism > 0.5	–	NA	NA	NA
Patients with anterior and posterior segment conditions	NA	–	NA	NA
Patients with posterior segment conditions only	NA	–	NA	NA
Mean VA for controls with astigmatism ≤ 0.5 vs > 0.5 D	**	NA	NA	NA
Mean VA for patients vs controls	NA	NA	***	*
Mean VA for patients with anterior and posterior segments conditions vs posterior segment conditions alone	NA	–	NA	NA
RMSHO 3 mm vs 5 mm	***	***	NA	NA
RMSHO for patients vs controls	NA	NA	**	–
Association between RMSHO (3 mm) vs Age	**	–	NA	NA
Association between VA and RMSHO (3 mm)	–	–	NA	NA
Spherical aberration 3 mm vs 5 mm pupil	***	***	NA	NA
Spherical Aberrations (3 mm) vs Age	–	–	NA	NA
Spherical Aberrations (5 mm) vs Age	***	–	NA	NA
RMSHO vs CMT	–	–	NA	NA
Mean VA vs CMT	–	–	NA	NA
SD of VA vs CMT	–	**	NA	NA

Level of Significance: *** Strong significance *p* < 0.001; ** Moderate significance *p* < 0.05 to *p* > 0.001;

* Barely missed significance *p* < 0.1 to *p* > 0.05; – No statistical significance; NA Not Applicable.

**Table 6:** Summary statistics indicating statistical significance for induced aberrations.

Statistical Comparisons	Controls	Patients	Controls vs Patients	Age Balanced Controls Vs Patients
**Induced Aberrations**				
Mean VA with vs without induced aberrations	***	** to ***	NA	NA
Mean VA with induced X Coma for Patients vs Controls	NA	NA	NA	**
Mean VA with induced Y Coma for Patients vs Controls	NA	NA	NA	**
Slope of VA decrements with induced positive spherical aberrations vs Age	–	–	NA	NA
Slope of VA decrements with induced negative spherical aberrations vs Age	–	–	NA	NA
Slope of VA decrement for induced positive vs negative spherical aberrations	***	***	NA	NA

Level of Significance: *** Strong significance *p* < 0.001; ** Moderate significance *p* < 0.05 to *p* > 0.001;

* Barely missed significance *p* < 0.1 to *p* > 0.05; – No statistical significance; NA Not Applicable.

## Discussion

Using Maxwellian view projection and a 3 mm pupil, along with a small field of view, we found good VA in control subjects and most patients with anterior segment, posterior segment, or both anterior and posterior segment conditions. The control group provided a larger sample and a much wider age range than in our initial findings [27] or with the PVT2 that used an adaptive optics system with more precision to correct inherent wavefront aberrations [31]. In this study we replicated our previous control group findings that optical aberrations were significantly reduced for a 3 mm vs 5 mm pupil [13] with new data from patients who had anterior segment changes, posterior segment changes, or both. This study demonstrated that VA measurements were not influenced by age for any group tested, which could not be tested in our original studies due to a limited range of ages (Table 5). This methodology provides not only means but also SDs for individual subjects, which also were not influenced by age. The use of a cumulative Gaussian function to obtain the individual SD’s is based on the assumption that the noise in the measurements has a Gaussian distribution [55,56]. Further, alternative approaches such QUEST and Weibull function can be problematic in a patient population or with extreme aberrations because the prior odds are unknown. The Gaussian fits provide a fit to not only the center of the distribution but also the more extreme values, likely giving a better estimate of the variability than fitting near the central tendency only.

While the 3 mm pupil reduced the measured astigmatism compared with a 5 mm pupil, the 3 mm pupil did not overcome the effect of the astigmatism on VA. The larger control group in this study had a sufficient range of astigmatism to answer this question, indicating that future instruments require the expense and added complexity to correct astigmatism.

Our results indicated that the Maxwellian view projection with a 3 mm pupil can minimize the effects of the RMSHO on VA. The RMSHO was significantly reduced for a 3 mm vs 5 mm pupil. For every subject, both patients and controls, the RMSHO for the 5 mm pupil was larger compared to 3 mm pupil. This is consistent with previous studies showing that aberrations decrease with decreasing pupil size [13,15]. Several previous studies have reported that age-related changes in ocular structures contribute to an increase in ocular aberrations [14,15,17,57,58]. We found that the RMSHO significantly increased with age for a 3 mm pupil, but the VA did not.

The optical aberrations were significantly greater in patients compared to all control subjects, but not when considering only age-balanced controls (Fig 4). This result is not consistent with previous studies that reported a similar increase in higher order aberrations among subjects [41–43]; some of these studies include only patients with retinitis pigmentosa and diabetes, which lead to early cataracts [41–43] or have small effects for 3 mm pupils but did have a statistical significance for pseudophakic eyes for horizontal coma [43]. The disease-related increase in aberrations can be attributed to the corneal, lenticular, and vitreous changes which are known to increase the aberrations [59,60]. These findings highlight that real-world clinical populations often exhibit more pronounced optical aberrations that can influence the VA assessments, such as those measured with induced aberrations.

Spherical aberration was significantly reduced for a 3 mm vs 5 mm pupil, varying with age for the 5 mm pupil but not the 3 mm pupil. There was no significant change in VA with spherical aberrations for either controls or patients. It should be noted that the values found in older subjects with a 5 mm pupil were similar to the extreme magnitudes of the induced spherical aberrations used to test VA with a 3 mm pupil. We found that these larger spherical aberrations could lead to worse VA. Further, for both controls and patients a linear summation of the amount of spherical aberration described the VA data. The slope of this linear function differed somewhat among subjects, but did not differ as a group between patients vs controls. Likewise, this slope was not significantly associated with age. Positive spherical aberrations had a significantly larger reduction in VA measures compared to the negative spherical aberrations. This finding was due to neither artifact from other aberrations in our system nor accommodation as indicated by a lack of effect of age. Instead, our finding is consistent with the positive spherical aberration conditions leading to less transfer than the negative spherical aberration conditions of the high spatial frequency information needed to reach excellent VA.

Our findings shed light on the development of specialty lenses for myopia control or improvement of VA or extended depth of field with IOLs, which have led to a variety of findings with spherical aberrations and pupil size. Negative spherical aberration in a custom contact lens designed for a 5 mm pupil has been shown to slightly improve distance VA in children, with a 5 mm pupil [61] and in adults with a 4.5 mm pupil and simulated IOLs [53]. However, no improvement due to spherical aberration was found with single vision contact lenses and a pupil size of 6 mm [62]. Pupil apodization can be effective in improving VA, for a pupil diameter of 4 mm, but only for spherical aberration values ≤ 0 [63]. Clearly, there is a complex relation between spherical aberrations and pupil size that accounts for some of the differences among findings [53,54]. With a wider pupil, there would be more aberrations, which could distort letters and thus decrease VA. Simultaneously, there is the potential of more peripheral rays transferred to provide the high spatial frequency information needed to see small letters, e.g. logMAR < 0 or even −0.125 or −0.30. When a dilated pupil is used, then there is sufficient high spatial frequency information present to reach a logMAR = 0. If a criterion method is used to evaluate the extended depth of field, and VA of logMAR ≥ 0 is the criterion, then it is possible to reach this criterion over a wider range of defocus without ever necessarily reaching or improving on a logMAR < 0. Our results provide data for a 3 mm pupil, which corresponds to a pupil size similar to that achieved with presbyopia control [45]. The slopes of VA decrement as a function of spherical aberration can provide estimates of the decrease in VA with various amounts of spherical aberration.

Our findings that a worse VA is associated with even relatively small amounts of astigmatism has important implications for many aspects of current refractive practice. Specialty contact lenses, such as for myopia control, or IOLs are not routinely designed to correct for astigmatism or use with over-refraction of small amounts of astigmatism [61]. Among soft contact lenses wearers, a prevalence is found of about 47% of individuals astigmatism with ≥ 0.75 D [64], which in our study is sufficient to be associated with worse VA.

Our induced aberration data provides a starting point for predicting VA in a device that includes correction for only sphere and cylinder. If anterior segment disease is severe enough to cause large aberrations such as those induced, we would expect a decrement in VA. It is a study limitation that despite recruiting patients with complaints of cataract and/or posterior capsule opacification, we did not include any subjects with the extreme wavefront aberrations that we induced with adaptive optics. However, we did test three levels each for positive and negative spherical aberrations; while the slopes differed somewhat for the decrease in VA vs. magnitude of spherical aberration, an upper bound can be computed for decrease in VA, providing a first step in a model of VA (Table 6).

CMT did not differ significantly between controls and patients and showed no significant association with either the RMSHO or the mean VA. The subjects in this study adjusted the spherical correction, presumably to focus the target E on the plane of the photoreceptors even when this plane was elevated. Failure to focus on the photoreceptors in the presence of subretinal fluid that elevates the plane of photoreceptors could lead to hyperopic defocus. This can worsen the VA [49]. In patients, the variability measure of VA (SD) increased significantly with CMT, indicating that structural changes due to macular thickening could potentially influence the VA measurements. In the present study, the PVT allowed for ease of adjustment to correct sphere, and this was done subjectively. The resulting VAs were typically excellent. This study did not determine whether objective measurement of sphere provides the same VA as values from subjective methods, such as adjustment of the instrument or subjective refraction, and whether this holds for a wider sample of extremely thickened or distorted retinas. This is the key target population for our method, i.e., to improve measurement of VA in clinical trials and for management of patients with retinal disease.

Previously, we demonstrated that defocus achieved optically can be used to model the impact of experimental error on VA in clinical trials or patient management [49]. Our previous models demonstrate the expected decrease in VA when there is residual spherical error due to fluid elevation of the photoreceptor layer, and the impact when the habitual refraction is used. These models demonstrate how VA changes with photoreceptor layer elevation, whereas CMT could have no effect on VA. In the present study, which allowed for adjustment of sphere prior to VA testing, we found no effect of CMT on the mean VA. Further, the potential decrement in VA has clinical significance; for instance, 0.75 D of defocus can lead to variations in VA of 2–6 letters. This amount is sufficient to influence the judgement of non-inferiority, which can be only 3–5 letters. A larger amount is needed to cause variability sufficient to cloud the decision that is based on an improvement of 3 lines [1,2,6,65]. By building a more complete model of VA, including both mean and SD for the individual patient, then clinical trials can have confidence intervals that could improve decision making and greatly lower the cost of trials.

In summary, our findings suggest that projecting visual stimuli using a Maxwellian view with a 3 mm pupil effectively minimizes the impact of optical aberrations on VA measurements. However, correction of astigmatism remains necessary to ensure accurate assessment.

## Supporting information

S1 Text Individual subject information and ocular status. (DOCX)

S2 Text Visual acuity data of individual subjects for no induced aberrations condition. (DOCX)

S3 Text Visual acuity data of individual subjects for induced aberration conditions. (DOCX)
