# Supplementary material for: Visual acuity in controls and patients measured with Maxwellian view and a 3 mm pupil: Examining potential effects of inherent and induced aberrations
Source: PLoS One. 2026 Jun 29;21(6):e0352879. doi: 10.1371/journal.pone.0352879 (PMC13313377; doi:10.1371/journal.pone.0352879)
Supplement: S1 Text — (DOCX) [file pone.0352879.s001.docx]

**Table 1: Individual subject information and ocular status**

| Subject ID | Age (yr) | Anterior Segment Status (0= Control, 1= Patient) | Posterior Segment Status (0= Control, 1= Patient) | Central Macular Thickness (Microns) | Spherical Power (D) | Cylindrical Power (D) | RMSHO (Microns) |
| --- | --- | --- | --- | --- | --- | --- | --- |
| S1 | 44 | 0 | 0 | 298 | -3.59298 | -1.70924 | 0.084379 |
| S2 | 66 | 0 | 0 | 289 | 0.41307 | -1.16643 | 0.104359 |
| S3 | 32 | 0 | 0 | 256 | -1.39849 | -0.21177 | 0.036155 |
| S4 | 30 | 0 | 0 | 245 | -0.00982 | -0.36435 | 0.050383 |
| S5 | 30 | 0 | 0 | 254 | -0.11348 | -0.41266 | 0.063948 |
| S6 | 29 | 0 | 0 | 301 | -2.2952 | -0.48341 | 0.060719 |
| S7 | 27 | 0 | 1 | 301 | -0.57612 | -0.48255 | 0.038306 |
| S8 | 22 | 0 | 0 | 233 | -2.85878 | -1.09648 | 0.048346 |
| S9 | 60 | 0 | 0 | 280 | 0.715363 | -0.51101 | 0.074392 |
| S10 | 39 | 0 | 0 | 256 | 3.698624 | -2.52086 | 0.085808 |
| S11 | 78 | 1 | 1 | 286 | 0.75 | 0.00 | NA |
| S12 | 71 | 1 | 1 | 262 | 1.728802 | -1.07871 | 0.114958 |
| S13 | 62 | 0 | 1 | 259 | -2.1318 | -1.39186 | 0.145602 |
| S14 | 55 | 0 | 0 | 262 | -0.5788 | -0.55588 | 0.03893 |
| S15 | 57 | 0 | 0 | 261 | -3.30246 | -0.73552 | 0.059932 |
| S16 | 74 | 1 | 1 | 258 | 1.04119 | -1.93197 | 0.099845 |
| S17 | 75 | 0 | 1 | 331 | 4.166675 | -2.17462 | 0.151183 |
| S18 | 79 | 0 | 1 | 240 | -0.39335 | -0.85761 | 0.105928 |
| S19 | 70 | 0 | 1 | 321 | 1.404332 | -1.8533 | 0.121082 |
| S20 | 73 | 0 | 1 | 315 | 0.86881 | -1.68743 | 0.084121 |
| S21 | 78 | 0 | 0 | 272 | -1.16203 | -1.92428 | 0.229226 |
| S22 | 72 | 0 | 1 | 299 | 0.673611 | -0.94165 | 0.073275 |
| S23 | 68 | 0 | 0 | 287 | 0.462486 | -0.95084 | 0.061601 |
| S24 | 61 | 0 | 0 | 277 | 0.95927 | -0.72099 | 0.062654 |
| S25 | 50 | 1 | 1 | 306 | 0.178111 | -1.67105 | 0.134322 |
| S26 | 46 | 0 | 0 | 285 | -0.14588 | -0.34831 | 0.038196 |
| S27 | 37 | 0 | 0 | 274 | -0.7091 | -0.23002 | 0.06953 |
| S28 | 49 | 0 | 0 | 273 | -4.67534 | -0.22334 | 0.047624 |
| S29 | 44 | 0 | 0 | 257 | -2.03263 | -0.69014 | 0.041853 |
| S30 | 68 | 0 | 0 | 323 | 1.693984 | -0.81777 | 0.067702 |
| S31 | 64 | 1 | 1 | 276 | -0.85343 | -2.13724 | 0.186472 |
| S32 | 78 | 0 | 0 | 287 | -2.67538 | -1.37261 | 0.249309 |
| S33 | 63 | 1 | 1 | 287 | -3.11598 | -3.2135 | 0.151472 |
| S34 | 50 | 0 | 0 | 257 | 0.820338 | -0.93949 | 0.073382 |
| S35 | 75 | 0 | 0 | 320 | 2.116597 | -2.22979 | 0.166738 |
| S36 | 31 | 0 | 0 | 280 | -3.47954 | -0.35366 | 0.049916 |
| S37 | 41 | 0 | 0 | 252 | -2.39904 | -0.66529 | 0.066271 |
| S38 | 31 | 0 | 0 | 234 | -0.06962 | -0.32488 | 0.051045 |
| S39 | 26 | 0 | 0 | 290 | -0.01456 | -0.49081 | 0.037061 |
| S40 | 26 | 0 | 0 | 278 | -3.01349 | -2.62752 | 0.068692 |
| S41 | 31 | 0 | 0 | 271 | -1.60801 | -0.76758 | 0.061706 |
| S42 | 82 | 1 | 1 | 282 | -7.45452 | -1.65469 | 0.14457 |
| S43 | 70 | 1 | 1 | 495 | -0.03818 | -0.95994 | 0.269318 |
| S44 | 72 | 1 | 1 | 254 | -8.1 | -0.77 | 0.105435 |
| S45 | 36 | 0 | 0 | 289 | -3.18761 | -0.50657 | 0.04719 |
| S46 | 74 | 0 | 1 | 297 | -0.76891 | -1.32271 | 0.13952 |
| S47 | 53 | 0 | 0 | 249 | -4.1242 | -1.26379 | 0.077588 |
