# Supplementary material for: Visual acuity in controls and patients measured with Maxwellian view and a 3 mm pupil: Examining potential effects of inherent and induced aberrations
Source: PLoS One. 2026 Jun 29;21(6):e0352879. doi: 10.1371/journal.pone.0352879 (PMC13313377; doi:10.1371/journal.pone.0352879)
Supplement: S2 Text — (DOCX) [file pone.0352879.s002.docx]

**Table 1: Visual Acuity Data of individual subjects for no induced aberrations condition**

| **Subject ID** | **Fit Mean (MAR)** | **Fit SD** | **RMSE** |
| --- | --- | --- | --- |
| S1 | 1.100854 | 0.243406 | 0.064134 |
| S2 | 1.324017 | 0.268373 | 0.125812 |
| S3 | 0.892949 | 0.029338 | 0.125 |
| S4 | 0.639573 | 0.509432 | 0.127851 |
| S5 | 0.551778 | 0.723541 | 0.112155 |
| S6 | 0.751101 | 0.156068 | 0.020321 |
| S7 | 0.751781 | 0.093389 | 0.000301 |
| S8 | 0.938611 | 0.117635 | 0.001472 |
| S9 | 1.030669 | 0.506295 | 0.366481 |
| S10 | 1.80123 | 1.103827 | 0.310996 |
| S11 | 1.566305 | 0.818689 | 0.271104 |
| S12 | 1.446149 | 0.576636 | 0.307982 |
| S13 | 1.734496 | 0.050515 | 1.24E-16 |
| S14 | 0.982346 | 0.877396 | 0.386405 |
| S15 | 1.153426 | 0.11774 | 0.249999 |
| S16 | 1.447577 | 0.235372 | 0.001462 |
| S17 | 3.206145 | 1.535739 | 0.218566 |
| S18 | 1.510694 | 0.669118 | 0.011705 |
| S19 | 1.0623 | 0.390889 | 0.162592 |
| S20 | 1.844909 | 0.601658 | 0.155084 |
| S21 | 1.516409 | 0.478931 | 0.260058 |
| S22 | 1.209368 | 0.11769 | 0.000141 |
| S23 | 0.831356 | 0.237409 | 0.070028 |
| S24 | 2.27603 | 1.327644 | 0.342168 |
| S25 | 0.972566 | 0.187997 | 0.266877 |
| S26 | 0.618381 | 0.483306 | 0.178024 |
| S27 | 0.587974 | 0.545718 | 0.241865 |
| S28 | 0.823993 | 0.030605 | 0.176777 |
| S29 | 0.476153 | 0.145945 | 0.004369 |
| S30 | 0.816673 | 0.752568 | 0.265649 |
| S31 | 1.322138 | 0.049428 | 5.61E-16 |
| S32 | 1.172866 | 1.085152 | 0.249171 |
| S33 | 1.054835 | 0.555445 | 0.179721 |
| S34 | 0.476171 | 0.145981 | 0.125055 |
| S35 | 0.548651 | 0.542978 | 0.173818 |
| S36 | 1.090959 | 0.025144 | 1.8E-15 |
| S37 | 0.932574 | 1.575947 | 0.634724 |
| S38 | 0.506587 | 0.37543 | 0.184691 |
| S39 | 1.052039 | 0.032559 | 0.125 |
| S40 | 1.475815 | 0.783464 | 0.214638 |
| S41 | 0.694235 | 0.144977 | 0.125676 |
| S42 | 1.343038 | 0.047149 | 9.04E-16 |
| S43 | 2.226872 | 2.072937 | 0.312033 |
| S44 | 1.427487 | 0.51596 | 0.061471 |
| S45 | 0.985079 | 0.396142 | 0.235416 |
| S46 | 0.737217 | 0.71885 | 0.283756 |
| S47 | 1.633179 | 1.388929 | 0.287684 |
