# Supplementary material for: Visual acuity in controls and patients measured with Maxwellian view and a 3 mm pupil: Examining potential effects of inherent and induced aberrations
Source: PLoS One. 2026 Jun 29;21(6):e0352879. doi: 10.1371/journal.pone.0352879 (PMC13313377; doi:10.1371/journal.pone.0352879)
Supplement: S3 Text — (DOCX) [file pone.0352879.s003.docx]

**Table 1: Visual Acuity data of individual subjects for induced aberration conditions**

| **Subject_ID** | **FitMean (MAR)** | **FitSD** | **RMSE** | **Order** |
| --- | --- | --- | --- | --- |
| S1 | 1.100854 | 0.243406 | 0.064134 | 1 |
| S1 | 2.004879 | 0.721519 | 0.060282 | 2 |
| S1 | 1.431506 | 1.84437 | 0.347899 | 3 |
| S1 | 2.174885 | 1.430817 | 0.17127 | 4 |
| S1 | 2.074195 | 1.690281 | 0.370827 | 5 |
| S1 | 4.234509 | 2.582339 | 0.399189 | 6 |
| S1 | 1.64074 | 0.47364 | 0.275635 | 7 |
| S1 | 3.052802 | 1.914138 | 0.203372 | 8 |
| S1 | 2.06753 | 0.956608 | 0.200401 | 9 |
| S2 | 1.324017 | 0.268373 | 0.125812 | 1 |
| S2 | 2.035319 | 1.282685 | 0.377064 | 2 |
| S2 | 1.887183 | 1.152317 | 0.087628 | 3 |
| S2 | 1.896541 | 0.354352 | 0.029857 | 4 |
| S2 | 1.054414 | 0.304591 | 0.304585 | 5 |
| S2 | 2.741677 | 1.752473 | 0.256265 | 6 |
| S2 | 2.293524 | 1.469344 | 0.129067 | 7 |
| S2 | 3.385684 | 2.747425 | 0.146932 | 8 |
| S2 | 2.998393 | 1.106526 | 0.016728 | 9 |
| S3 | 0.892949 | 0.029338 | 0.125 | 1 |
| S3 | 2.998393 | 1.106526 | 0.016728 | 2 |
| S3 | 1.37618 | 0.920319 | 0.164952 | 3 |
| S3 | 1.36801 | 0.847563 | 0.059912 | 4 |
| S3 | 1.794719 | 0.601723 | 0.042147 | 5 |
| S3 | 1.745317 | 1.392349 | 0.32172 | 6 |
| S3 | 1.503913 | 0.320591 | 0.124612 | 7 |
| S3 | 2.880864 | 1.985663 | 0.187464 | 8 |
| S3 | 2.351008 | 0.374948 | 0.093512 | 9 |
| S4 | 0.639573 | 0.509432 | 0.127851 | 1 |
| S4 | 2.095382 | 0.538291 | 0.120853 | 2 |
| S4 | 1.816684 | 1.592872 | 0.222535 | 3 |
| S4 | 2.828562 | 1.96529 | 0.307156 | 4 |
| S4 | 1.851433 | 1.146507 | 0.335386 | 5 |
| S4 | 2.060848 | 1.154442 | 0.166834 | 6 |
| S4 | 2.313434 | 0.715392 | 0.084328 | 7 |
| S4 | 2.084536 | 0.850048 | 0.136851 | 8 |
| S4 | 2.826971 | 2.297554 | 0.19462 | 9 |
| S5 | 0.551778 | 0.723541 | 0.112155 | 1 |
| S5 | 2.82146 | 0.958521 | 0.003956 | 2 |
| S5 | 3.150472 | 1.21671 | 0.03734 | 3 |
| S5 | 2.319743 | 1.661671 | 0.064061 | 4 |
| S5 | 1.510061 | 0.647725 | 0.331304 | 5 |
| S5 | 2.005175 | 0.722001 | 0.138775 | 6 |
| S5 | 1.535745 | 0.506925 | 0.088118 | 7 |
| S5 | 2.998393 | 1.106526 | 0.016728 | 8 |
| S5 | 1.582959 | 1.581319 | 0.266373 | 9 |
| S6 | 0.751101 | 0.156068 | 0.020321 | 1 |
| S6 | 1.641989 | 1.712708 | 0.353587 | 2 |
| S6 | 1.025901 | 0.138872 | 0.129172 | 3 |
| S6 | 1.758725 | 0.994581 | 0.546636 | 4 |
| S6 | 1.311371 | 0.891333 | 0.190295 | 5 |
| S6 | 2.651304 | 1.371811 | 0.025743 | 6 |
| S6 | 2.132708 | 0.73929 | 0.320269 | 7 |
| S6 | 3.45173 | 1.341442 | 0.094229 | 8 |
| S6 | 1.894883 | 1.181815 | 0.151481 | 9 |
| S7 | 0.751781 | 0.093389 | 0.000301 | 1 |
| S7 | 1.730791 | 0.771737 | 0.281473 | 2 |
| S7 | 0.734206 | 0.518237 | 0.289561 | 3 |
| S7 | 1.452122 | 0.249409 | 0.248088 | 4 |
| S7 | 1.074131 | 0.187542 | 0.124713 | 5 |
| S7 | 1.510061 | 0.647725 | 0.331304 | 6 |
| S7 | 1.715675 | 0.3729 | 0.216736 | 7 |
| S7 | 2.651304 | 1.371811 | 0.025743 | 8 |
| S7 | 1.789829 | 0.062093 | 0.176777 | 9 |
| S8 | 0.938611 | 0.117635 | 0.001472 | 1 |
| S8 | 1.645603 | 1.089762 | 0.269899 | 2 |
| S8 | 1.242496 | 0.148115 | 0.124392 | 3 |
| S8 | 1.280579 | 0.697861 | 0.195253 | 4 |
| S8 | 1.016713 | 0.320624 | 0.31522 | 5 |
| S8 | 3.92003 | 3.606304 | 0.502598 | 6 |
| S8 | 1.447298 | 0.379912 | 0.081619 | 7 |
| S8 | 4.150832 | 2.71472 | 0.312197 | 8 |
| S8 | 1.309995 | 0.833012 | 0.356896 | 9 |
| S9 | 1.030669 | 0.506295 | 0.366481 | 1 |
| S9 | 1.468686 | 0.319836 | 0.235942 | 2 |
| S9 | 1.212512 | 0.554473 | 0.112755 | 3 |
| S9 | 1.559577 | 0.235482 | 0.124992 | 4 |
| S9 | 2.011963 | 0.898612 | 0.092653 | 5 |
| S9 | 1.742671 | 0.076172 | 0.125 | 6 |
| S9 | 1.332663 | 0.955794 | 0.334051 | 7 |
| S9 | 3.206138 | 1.535666 | 0.243905 | 8 |
| S9 | 2.651304 | 1.371811 | 0.025743 | 9 |
| S10 | 1.80123 | 1.103827 | 0.310996 | 1 |
| S10 | 3.040096 | 1.461791 | 0.320542 | 2 |
| S10 | 2.361998 | 2.268435 | 0.179661 | 3 |
| S10 | 2.998393 | 1.106526 | 0.016728 | 4 |
| S10 | 2.676106 | 1.200194 | 0.222148 | 5 |
| S10 | 2.501643 | 1.160355 | 0.006885 | 6 |
| S10 | 3.298027 | 1.296087 | 0.063815 | 7 |
| S10 | 2.277182 | 1.752354 | 0.351252 | 8 |
| S10 | 2.539447 | 1.145878 | 0.073845 | 9 |
| S11 | 1.566305 | 0.818689 | 0.271104 | 1 |
| S11 | 3.947811 | 4.07985 | 0.215871 | 2 |
| S11 | 2.331417 | 2.567179 | 0.216233 | 3 |
| S11 | 4.419475 | 4.887975 | 0.41996 | 4 |
| S11 | 2.648895 | 3.794443 | 0.420744 | 5 |
| S11 | 2.227139 | 0.56863 | 0.294014 | 6 |
| S11 | 2.17126 | 1.815456 | 0.455552 | 7 |
| S11 | 3.98692 | 2.66783 | 0.407512 | 8 |
| S11 | 2.438623 | 1.632981 | 0.296544 | 9 |
| S12 | 1.446149 | 0.576636 | 0.307982 | 1 |
| S12 | 2.138547 | 0.55307 | 0.182218 | 2 |
| S12 | 2.82146 | 0.958521 | 0.003956 | 3 |
| S12 | 1.924215 | 1.605194 | 0.522661 | 4 |
| S12 | 2.501643 | 1.160355 | 0.006885 | 5 |
| S12 | 2.770522 | 1.307446 | 0.27649 | 6 |
| S12 | 1.799311 | 0.854858 | 0.374497 | 7 |
| S12 | 5.659655 | 5.770099 | 0.277602 | 8 |
| S12 | 2.781859 | 1.533668 | 0.052971 | 9 |
| S13 | 1.734496 | 0.050515 | 1.24E-16 | 1 |
| S13 | 1.710663 | 1.575954 | 0.158239 | 2 |
| S13 | 2.226651 | 1.404733 | 0.091981 | 3 |
| S13 | 4.407433 | 3.209531 | 0.337586 | 4 |
| S13 | 2.147183 | 1.33465 | 0.083719 | 5 |
| S13 | 2.396923 | 3.846248 | 0.253552 | 6 |
| S13 | 1.865609 | 0.74327 | 0.139344 | 7 |
| S13 | 2.856428 | 5.202222 | 0.150283 | 8 |
| S13 | 1.8941 | 1.720137 | 0.377475 | 9 |
| S14 | 0.982346 | 0.877396 | 0.386405 | 1 |
| S14 | 2.718943 | 1.530583 | 0.271574 | 2 |
| S14 | 2.220774 | 0.760431 | 0.087077 | 3 |
| S14 | 2.501643 | 1.160355 | 0.006885 | 4 |
| S14 | 1.876007 | 0.905433 | 0.053378 | 5 |
| S14 | 2.82146 | 0.958521 | 0.003956 | 6 |
| S14 | 1.883571 | 1.524979 | 0.564449 | 7 |
| S14 | 3.985195 | 1.823465 | 0.065397 | 8 |
| S14 | 2.693773 | 0.899717 | 0.082493 | 9 |
| S15 | 1.153426 | 0.11774 | 0.249999 | 1 |
| S15 | 1.516934 | 1.236364 | 0.170938 | 2 |
| S15 | 1.821794 | 0.719883 | 0.236548 | 3 |
| S15 | 1.587516 | 0.53281 | 0.289996 | 4 |
| S15 | 1.986242 | 0.709612 | 0.076325 | 5 |
| S15 | 2.796541 | 1.212759 | 0.162891 | 6 |
| S15 | 1.562745 | 0.25272 | 0.117137 | 7 |
| S15 | 1.280034 | 0.347782 | 0.196955 | 8 |
| S15 | 1.78785 | 0.540212 | 0.090733 | 9 |
| S16 | 1.447577 | 0.235372 | 0.001462 | 1 |
| S16 | 2.651304 | 1.371811 | 0.025743 | 2 |
| S16 | 2.938185 | 1.593737 | 0.242218 | 3 |
| S16 | 3.637452 | 2.422204 | 0.232989 | 4 |
| S16 | 1.834822 | 0.624359 | 0.306146 | 5 |
| S16 | 3.556318 | 2.018492 | 0.156709 | 6 |
| S16 | 1.242554 | 0.975082 | 0.538576 | 7 |
| S16 | 3.715463 | 3.168944 | 0.209307 | 8 |
| S16 | 2.586523 | 1.251612 | 0.203313 | 9 |
| S17 | 4.266115 | 0.189974 | 0.125 | 2 |
| S17 | 3.765464 | 2.857623 | 0.190188 | 3 |
| S17 | 3.616766 | 2.122347 | 0.184559 | 4 |
| S17 | 3.535008 | 2.025031 | 0.265957 | 5 |
| S17 | 2.82146 | 0.958521 | 0.003956 | 6 |
| S17 | 5.229469 | 1.809087 | 0.241011 | 7 |
| S17 | 2.339576 | 0.673719 | 0.205219 | 8 |
| S17 | 5.847107 | 1.828332 | 0.230934 | 9 |
| S17 | 3.206145 | 1.535739 | 0.218566 | 1 |
| S19 | 1.0623 | 0.390889 | 0.162592 | 1 |
| S19 | 2.559805 | 1.515378 | 0.122752 | 2 |
| S19 | 2.308241 | 2.023332 | 0.476444 | 3 |
| S19 | 2.752496 | 1.60537 | 0.085942 | 4 |
| S19 | 3.007179 | 1.120377 | 0.139755 | 5 |
| S19 | 3.233271 | 1.397269 | 0.073109 | 6 |
| S19 | 2.581675 | 0.49676 | 0.166285 | 7 |
| S19 | 4.045997 | 0.162962 | 2.22E-16 | 8 |
| S19 | 3.530588 | 1.566536 | 0.017182 | 9 |
| S20 | 1.844909 | 0.601658 | 0.155084 | 1 |
| S20 | 4.205565 | 3.315963 | 0.11167 | 2 |
| S20 | 4.27642 | 3.034447 | 0.347152 | 3 |
| S20 | 3.393946 | 4.821941 | 0.214344 | 4 |
| S20 | 2.670994 | 1.412207 | 0.0884 | 5 |
| S20 | 3.356608 | 1.192162 | 0.372868 | 7 |
| S20 | 5.912941 | 2.563534 | 0.3942 | 8 |
| S20 | 4.266115 | 0.189974 | 0.125 | 9 |
| S20 | 6.450906 | 5.08538 | 0.395558 | 6 |
| S23 | 0.831356 | 0.237409 | 0.070028 | 1 |
| S23 | 2.150058 | 0.993569 | 0.231756 | 2 |
| S23 | 1.587783 | 1.457146 | 0.114056 | 3 |
| S23 | 1.451337 | 0.847862 | 0.428814 | 4 |
| S23 | 1.510161 | 2.096616 | 0.579213 | 5 |
| S23 | 3.17648 | 1.835691 | 0.161581 | 6 |
| S23 | 1.742903 | 0.45662 | 0.154535 | 7 |
| S23 | 1.573108 | 0.722196 | 0.129535 | 9 |
| S23 | 3.039501 | 1.773652 | 0.174321 | 8 |
| S24 | 2.27603 | 1.327644 | 0.342168 | 1 |
| S24 | 3.150472 | 1.21671 | 0.03734 | 2 |
| S24 | 1.718415 | 0.373514 | 0.062498 | 3 |
| S24 | 2.072609 | 1.137476 | 0.02773 | 4 |
| S24 | 2.56019 | 1.92593 | 0.484738 | 5 |
| S24 | 1.787005 | 2.556942 | 0.427039 | 6 |
| S24 | 2.081192 | 1.156284 | 0.374656 | 7 |
| S24 | 3.502771 | 3.161005 | 0.276233 | 8 |
| S24 | 1.564523 | 0.369894 | 0.07141 | 9 |
| S25 | 0.972566 | 0.187997 | 0.266877 | 1 |
| S25 | 1.366065 | 1.845017 | 0.215874 | 2 |
| S25 | 1.209422 | 0.117739 | 0.30618 | 3 |
| S25 | 3.298027 | 1.296087 | 0.063815 | 4 |
| S25 | 1.705255 | 0.56729 | 0.073037 | 5 |
| S25 | 2.686353 | 0.795951 | 0.115999 | 6 |
| S25 | 2.19557 | 1.396099 | 0.197902 | 7 |
| S25 | 3.303862 | 2.452234 | 0.144247 | 8 |
| S25 | 3.021007 | 2.049066 | 0.378707 | 9 |
| S26 | 0.618381 | 0.483306 | 0.178024 | 1 |
| S26 | 4.350989 | 0.172575 | 0.139754 | 2 |
| S26 | 2.883802 | 1.035874 | 0.172779 | 3 |
| S26 | 1.112613 | 0.175542 | 0.259677 | 4 |
| S26 | 4.197539 | 0.145978 | 4.97E-16 | 5 |
| S26 | 3.764615 | 2.854855 | 0.179708 | 6 |
| S26 | 2.114937 | 0.049019 | 3.2E-16 | 7 |
| S26 | 3.821401 | 1.849897 | 0.096517 | 8 |
| S26 | 1.308656 | 0.777944 | 0.349735 | 9 |
| S27 | 0.587974 | 0.545718 | 0.241865 | 1 |
| S27 | 2.887091 | 1.790552 | 0.052873 | 2 |
| S27 | 3.484723 | 5.057826 | 0.406733 | 4 |
| S27 | 0.916644 | 0.912031 | 0.261384 | 5 |
| S27 | 2.218608 | 1.065167 | 0.367941 | 6 |
| S27 | 1.653559 | 0.420729 | 0.014967 | 7 |
| S27 | 3.419855 | 3.864894 | 0.241774 | 8 |
| S27 | 2.651288 | 1.371797 | 0.067594 | 9 |
| S27 | 1.48027 | 1.178654 | 0.321283 | 3 |
| S28 | 0.823993 | 0.030605 | 0.176777 | 1 |
| S28 | 2.287246 | 1.547448 | 0.200987 | 2 |
| S28 | 2.362542 | 0.187493 | 0.124713 | 3 |
| S28 | 2.202515 | 3.338462 | 0.241977 | 4 |
| S28 | 1.725369 | 0.673168 | 0.028951 | 5 |
| S28 | 3.212822 | 1.493807 | 0.576976 | 6 |
| S28 | 1.212582 | 0.618764 | 0.133125 | 7 |
| S28 | 4.355177 | 1.347779 | 0.230337 | 8 |
| S28 | 1.322138 | 0.049428 | 5.61E-16 | 9 |
| S29 | 0.476153 | 0.145945 | 0.004369 | 1 |
| S29 | 3.381429 | 1.501902 | 0.040945 | 2 |
| S29 | 1.500027 | 1.537202 | 0.199977 | 3 |
| S29 | 2.82146 | 0.958521 | 0.003956 | 4 |
| S29 | 1.047026 | 0.243747 | 0.063955 | 5 |
| S29 | 2.232759 | 3.717656 | 0.201583 | 6 |
| S29 | 1.140988 | 0.308552 | 0.122054 | 7 |
| S29 | 3.317896 | 1.912163 | 0.236633 | 8 |
| S29 | 1.278956 | 0.469418 | 0.164065 | 9 |
| S30 | 0.816673 | 0.752568 | 0.265649 | 1 |
| S30 | 3.099564 | 2.977997 | 0.230519 | 2 |
| S30 | 1.977376 | 0.545305 | 0.121852 | 3 |
| S30 | 2.087939 | 1.176028 | 0.2577 | 4 |
| S30 | 2.781859 | 1.533668 | 0.052971 | 5 |
| S30 | 3.079629 | 0.876872 | 0.160003 | 6 |
| S30 | 1.343053 | 0.460856 | 0.080671 | 7 |
| S30 | 4.004575 | 2.078228 | 0.162364 | 8 |
| S30 | 1.570091 | 0.770161 | 0.268306 | 9 |
| S41 | 1.0623 | 0.390889 | 0.162592 | 5 |
| S32 | 1.172866 | 1.085152 | 0.249171 | 1 |
| S32 | 2.056176 | 0.60013 | 0.148232 | 2 |
| S32 | 1.719004 | 0.368803 | 0.010316 | 3 |
| S32 | 1.395287 | 0.510274 | 0.231519 | 4 |
| S32 | 1.559528 | 0.234875 | 0.006163 | 5 |
| S32 | 2.248495 | 1.162159 | 0.343281 | 6 |
| S32 | 1.87897 | 0.240612 | 0.123362 | 7 |
| S32 | 4.039735 | 0.222772 | 0.125 | 8 |
| S32 | 2.594181 | 1.337772 | 0.113188 | 9 |
| S33 | 1.054835 | 0.555445 | 0.179721 | 1 |
| S33 | 1.742671 | 0.076172 | 0.125 | 2 |
| S33 | 1.419728 | 1.363633 | 0.262426 | 3 |
| S33 | 1.994161 | 0.370083 | 0.071343 | 4 |
| S33 | 2.651304 | 1.371811 | 0.025743 | 5 |
| S33 | 3.627182 | 1.335757 | 0.12678 | 6 |
| S33 | 1.503745 | 0.319446 | 0.06212 | 7 |
| S33 | 4.250416 | 2.925496 | 0.243067 | 8 |
| S33 | 3.023425 | 2.742897 | 0.23875 | 9 |
| S34 | 0.476171 | 0.145981 | 0.125055 | 1 |
| S34 | 1.405972 | 1.345422 | 0.377055 | 2 |
| S34 | 0.722098 | 1.04731 | 0.298149 | 3 |
| S34 | 0.834444 | 0.897092 | 0.487539 | 4 |
| S34 | 0.939508 | 0.120298 | 0.123362 | 5 |
| S34 | 2.334921 | 1.218721 | 0.311561 | 6 |
| S34 | 1.181581 | 0.16036 | 0.176502 | 7 |
| S34 | 2.007336 | 2.234645 | 0.234163 | 8 |
| S34 | 1.414405 | 0.733109 | 0.330186 | 9 |
| S35 | 0.548651 | 0.542978 | 0.173818 | 1 |
| S35 | 2.186535 | 0.754535 | 0.182662 | 2 |
| S35 | 1.013998 | 3.489315 | 0.437001 | 3 |
| S35 | 2.957948 | 1.880208 | 0.302546 | 4 |
| S35 | 2.781859 | 1.533668 | 0.052971 | 5 |
| S35 | 1.986242 | 0.709612 | 0.076325 | 6 |
| S35 | 2.362363 | 0.18793 | 0.225104 | 7 |
| S35 | 2.651304 | 1.371811 | 0.025743 | 8 |
| S35 | 3.531548 | 3.214748 | 0.15751 | 9 |
| S36 | 1.090959 | 0.025144 | 1.8E-15 | 1 |
| S36 | 2.098704 | 1.640384 | 0.178399 | 2 |
| S36 | 1.179275 | 0.756011 | 0.485454 | 3 |
| S36 | 1.358903 | 1.298788 | 0.380809 | 4 |
| S36 | 1.492653 | 0.858646 | 0.383114 | 5 |
| S36 | 1.887183 | 1.152317 | 0.087628 | 6 |
| S36 | 1.360618 | 1.6231 | 0.226546 | 7 |
| S36 | 3.150472 | 1.21671 | 0.03734 | 8 |
| S36 | 2.998393 | 1.106526 | 0.016728 | 9 |
| S37 | 0.932574 | 1.575947 | 0.634724 | 1 |
| S37 | 3.150472 | 1.21671 | 0.03734 | 2 |
| S37 | 2.501643 | 1.160355 | 0.006885 | 3 |
| S37 | 1.259964 | 1.338489 | 0.412358 | 4 |
| S37 | 0.789883 | 1.313383 | 0.322254 | 5 |
| S37 | 0.898158 | 0.482057 | 0.475058 | 6 |
| S37 | 0.692605 | 0.500169 | 0.178115 | 7 |
| S37 | 1.016208 | 0.318811 | 0.19226 | 8 |
| S37 | 0.922498 | 0.300835 | 0.155084 | 9 |
| S38 | 0.506587 | 0.37543 | 0.184691 | 1 |
| S38 | 1.655203 | 1.266662 | 0.254616 | 2 |
| S38 | 1.419224 | 0.646924 | 0.112979 | 3 |
| S38 | 1.808559 | 1.804511 | 0.18295 | 4 |
| S38 | 2.501643 | 1.160355 | 0.006885 | 5 |
| S38 | 3.14169 | 4.778519 | 0.360609 | 6 |
| S38 | 1.763463 | 0.06681 | 0.25 | 7 |
| S38 | 2.88044 | 2.820146 | 0.375383 | 8 |
| S38 | 1.767463 | 0.390209 | 0.274732 | 9 |
| S42 | 1.343038 | 0.047149 | 9.04E-16 | 1 |
| S42 | 2.692269 | 1.060323 | 0.414053 | 3 |
| S42 | 1.574422 | 0.627531 | 0.307802 | 5 |
| S42 | 5.226839 | 5.569577 | 0.354233 | 6 |
| S42 | 2.623671 | 3.306819 | 0.172053 | 7 |
| S42 | 4.761217 | 7.214534 | 0.479848 | 8 |
| S42 | 3.101663 | 2.763314 | 0.234318 | 9 |
| S42 | 1.786796 | 1.328423 | 0.291712 | 4 |
| S42 | 4.470983 | 0.152113 | 0.139754 | 2 |
| S39 | 1.052039 | 0.032559 | 0.125 | 1 |
| S39 | 2.2063 | 1.751947 | 0.322136 | 2 |
| S39 | 1.7184 | 0.071053 | 0.125 | 3 |
| S39 | 1.63461 | 1.56331 | 0.276639 | 4 |
| S39 | 1.877282 | 0.714512 | 0.07479 | 5 |
| S39 | 2.501643 | 1.160355 | 0.006885 | 6 |
| S39 | 1.309366 | 0.400342 | 0.371585 | 7 |
| S39 | 4.166814 | 2.112045 | 0.249072 | 8 |
| S39 | 1.896541 | 0.354352 | 0.029857 | 9 |
| S40 | 1.475815 | 0.783464 | 0.214638 | 1 |
| S40 | 4.518889 | 2.637383 | 0.328066 | 2 |
| S40 | 1.280187 | 0.620065 | 0.461577 | 3 |
| S40 | 2.543314 | 1.411626 | 0.193489 | 4 |
| S40 | 2.135149 | 0.541436 | 0.287892 | 5 |
| S40 | 1.488071 | 1.613968 | 0.281726 | 6 |
| S40 | 2.370732 | 1.296899 | 0.118683 | 7 |
| S40 | 2.922358 | 3.056295 | 0.355873 | 8 |
| S40 | 3.020162 | 1.926171 | 0.06802 | 9 |
| S41 | 0.694235 | 0.144977 | 0.125676 | 1 |
| S41 | 1.459749 | 0.863654 | 0.291104 | 2 |
| S41 | 1.07805 | 0.823504 | 0.651335 | 3 |
| S41 | 1.673851 | 0.066048 | 0.1875 | 4 |
| S41 | 2.177849 | 0.985827 | 0.150675 | 6 |
| S41 | 1.2383 | 0.304866 | 0.100333 | 7 |
| S41 | 1.367297 | 1.142004 | 0.234461 | 9 |
| S41 | 2.243612 | 1.852048 | 0.258739 | 8 |
| S43 | 2.226872 | 2.072937 | 0.312033 | 1 |
| S43 | 2.376991 | 1.056558 | 0.263213 | 2 |
| S43 | 1.609984 | 1.178064 | 0.136303 | 3 |
| S43 | 1.802633 | 1.29531 | 0.557356 | 4 |
| S43 | 4.149584 | 3.474597 | 0.370865 | 5 |
| S43 | 1.877313 | 1.671663 | 0.227125 | 6 |
| S43 | 1.943808 | 1.038928 | 0.341114 | 7 |
| S43 | 3.150472 | 1.21671 | 0.03734 | 8 |
| S43 | 2.028529 | 0.77178 | 0.253306 | 9 |
| S45 | 0.985079 | 0.396142 | 0.235416 | 1 |
| S45 | 2.998393 | 1.106526 | 0.016728 | 2 |
| S45 | 1.366141 | 1.052608 | 0.319565 | 3 |
| S45 | 1.601217 | 0.390306 | 0.231675 | 4 |
| S45 | 1.68317 | 0.721445 | 0.114814 | 5 |
| S45 | 2.595981 | 1.144893 | 0.315637 | 6 |
| S45 | 2.199836 | 1.655941 | 0.495346 | 8 |
| S45 | 1.801738 | 0.45399 | 0.052512 | 7 |
| S45 | 2.82146 | 0.958521 | 0.003956 | 9 |
| S46 | 0.737217 | 0.71885 | 0.283756 | 1 |
| S46 | 1.66687 | 1.101325 | 0.228729 | 2 |
| S46 | 1.853709 | 0.53684 | 0.127182 | 3 |
| S46 | 1.307316 | 0.387628 | 0.115709 | 4 |
| S46 | 1.767587 | 0.795371 | 0.056146 | 5 |
| S46 | 1.662712 | 0.474819 | 0.070028 | 6 |
| S46 | 1.181575 | 0.160313 | 0.124612 | 7 |
| S46 | 1.7184 | 0.054536 | 1.67E-15 | 8 |
| S46 | 1.800865 | 0.38862 | 0.129602 | 9 |
| S47 | 1.633179 | 1.388929 | 0.287684 | 1 |
| S47 | 2.594931 | 1.803361 | 0.270707 | 2 |
| S47 | 3.686473 | 1.591863 | 0.131934 | 3 |
| S47 | 2.118639 | 0.803556 | 0.33461 | 4 |
| S47 | 1.654219 | 0.906071 | 0.212746 | 5 |
| S47 | 2.594715 | 0.71988 | 0.025843 | 8 |
| S47 | 2.501643 | 1.160355 | 0.006885 | 7 |
| S47 | 2.226651 | 1.404733 | 0.091981 | 6 |
| S47 | 2.651304 | 1.371811 | 0.025743 | 9 |
| *Order 1: No Induced Aberrations, Order 2: Y Coma +0.49, Order 3: Y Coma -0.59,  Order 4: X Coma -0.51, Order 5: X Coma +0.63, Order 6: Spherical +0.27, Order 7: Spherical -0.23, Order 8: Spherical +0.39, Order 9: Spherical -0.32 | | | | |
